# Tet-mediated DNA demethylation regulates specification of hematopoietic stem and progenitor cells during mammalian embryogenesis

**DOI:** 10.1126/sciadv.abm3470

**Published:** 2022-03-02

**Authors:** Liyang Ma, Qin Tang, Xin Gao, Joun Lee, Run Lei, Masako Suzuki, Deyou Zheng, Keisuke Ito, Paul S. Frenette, Meelad M. Dawlaty

**Affiliations:** 1 Ruth L. and David S. Gottesman Institute for Stem Cell and Regenerative Medicine Research, Albert Einstein College of Medicine, 1301 Morris Park Ave., Bronx, NY 10461, USA.; 2 Department of Genetics, Albert Einstein College of Medicine, 1300 Morris Park Ave., Bronx, NY 10461, USA.; 3 Department of Developmental and Molecular Biology, Albert Einstein College of Medicine, 1300 Morris Park Ave., Bronx, NY 10461, USA.; 4 Department of Cell Biology, Albert Einstein College of Medicine, 1300 Morris Park Ave., Bronx, NY 10461, USA.; 5 Departments of Neurology and Neuroscience, Albert Einstein College of Medicine, 1300 Morris Park Ave., Bronx, NY 10461, USA.; 6 Department of Medicine, Albert Einstein College of Medicine, 1300 Morris Park Ave., Bronx, NY 10461, USA.

## Abstract

Ten-eleven translocation (Tet) enzymes promote DNA demethylation by oxidizing 5-methylcytosine. They are expressed during development and are essential for mouse gastrulation. However, their postgastrulation functions are not well established. We find that global or endothelial-specific loss of all three Tet enzymes immediately after gastrulation leads to reduced number of hematopoietic stem and progenitor cells (HSPCs) and lethality in mid-gestation mouse embryos. This is due to defects in specification of HSPCs from endothelial cells (ECs) that compromise primitive and definitive hematopoiesis. Mechanistically, loss of Tet enzymes in ECs led to hypermethylation and down-regulation of NFκB1 and master hematopoietic transcription factors (Gata1/2, Runx1, and Gfi1b). Restoring Tet catalytic activity or overexpression of these factors in Tet-deficient ECs rescued hematopoiesis defects. This establishes Tet enzymes as activators of hematopoiesis programs in ECs for specification of HSPCs during embryogenesis, which is distinct from their roles in adult hematopoiesis, with implications in deriving HSPCs from pluripotent cells.

## Teaser

Tet enzymes promote emergence of hematopoietic progenitor cells during embryogenesis by activating hematopoietic genes.

## INTRODUCTION

DNA methylation [5-methylcytosine (5mC)] is a major modification in our genome that silences genes. Its levels change dynamically during development to activate or repress genes critical for lineage specification and organogenesis (*1*). While DNA methylation is achieved by the DNA methyltransferase enzymes (Dnmts), DNA demethylation is carried out by the DNA demethylase enzymes Tet1, Tet2, and Tet3 (*2*). Tet enzymes promote DNA demethylation and gene activation by iterative conversion of 5-methylcytosine (5mC) to 5-hydroxy-methylcytosine (5hmC), 5-formylcytosine, and 5-carboxylcytosine (*3*–*5*). These modified bases are either actively removed from the genome to promote active demethylation or they inhibit Dnmt1 and *Uhrf1* recruitment during replication to promote passive DNA demethylation (*6*).

Tet enzymes are dynamically expressed during embryogenesis and are required for development. Tet3 is highly expressed in the oocyte where it facilitates paternal genome demethylation, and deficiency of Tet3 in mice leads to perinatal lethality (*7*). Tet1 and Tet2 are expressed in the inner cell mass of developing blastocysts and in embryonic stem cells (ESCs) where they regulate pluripotency and differentiation programs (*8*, *9*). Individual or combined loss of Tet1 and Tet2 in ESCs does not block differentiation (*8*, *10*, *11*). Tet1-deficient mice are runted in 129/B6 background and lethal or sublethal in other genetic backgrounds (*10*, *12*, *13*). Tet2 is dispensable for development, and Tet2 knockout mice grow normally to adulthood but develop leukemia later in life (*14*, *15*). Combined deficiency of Tet1 and Tet2 leads to partial perinatal lethality with most of the embryos dying by mid-gestation and only a fraction surviving to adulthood (*11*). Likewise, combined loss of Tet1 and Tet3 leads to mid-gestation lethality with most of the embryos not developing beyond embryonic day 10.5 (E10.5) (*16*). These findings suggest that Tet enzymes partly compensate for each other during development. Consistently, loss of all three Tet enzymes compromises ESC pluripotency and arrests development at gastrulation (*17*, *18*). These findings suggest a critical requirement for Tet enzymes and DNA hydroxymethylation in early development. Although Tet enzymes are expressed in various lineages during mammalian postgastrulation development, owing to the early embryonic lethality of Tet triple knockout (TKO) mice, developmental functions of Tet enzymes and the absolute requirements of DNA hydroxylation in lineage specification and organogenesis have not been well defined. There are few reports on conditional lineage-specific loss of Tet enzymes during postgastrulation development. Cardiac-specific deletion of Tet2/3 in mice leads to defects in heart development and late gestation lethality (*19*). However, roles of Tet enzymes in other lineages where Tet enzymes are highly expressed, such as the neural and hematopoietic lineages, are poorly defined.

Hematopoiesis is a complex process that gives rise to all blood cell types in our body. Proper hematopoiesis is essential not only for robust functioning of our immune system but also for our overall physiology. Aberrant hematopoiesis can lead to many human diseases including hematological malignancies (*20*). During embryogenesis, hematopoiesis is initiated shortly after gastrulation. Early blood progenitor cells, mainly erythroid progenitors, emerge from the endothelial cells (ECs) in the yolk sac (YS) around E7.5 of mouse embryonic development. This process is termed primitive hematopoiesis (*21*, *22*). As embryogenesis proceeds, hematopoietic stem and progenitor cells (HSPCs) emerge from the endothelial lining of aortic vasculature involving the dorsal aorta in the aorta-gonad-mesonephros (AGM). This process is referred to as definitive hematopoiesis (*21*, *23*–*25*). The emerging HSPCs from the YS and AGM migrate and populate the fetal liver (FL) by E11.5. Later in development, they home into the bone marrow, which becomes the long-term site of hematopoiesis (*23*). During embryonic hematopoiesis, HSPCs are specified from ECs. Specialized ECs in the dorsal aorta, termed hemogenic ECs (HECs), which are CD31^+^ c-Kit^–^ Runx1^+^, emerge as clusters from the arterial endothelium and serve as precursors to HSPCs. This process is called endothelial-to-hematopoietic transition (*26*–*29*). Specification of ECs to HECs and subsequently to HSPCs is guided by timely expression of hematopoiesis-specific genes including members of the *Runx*, *Gata*, and *Gfi1* families of master transcription factors. Proper activation of these genes in ECs and HECs and their expression in HSPCs is essential for normal embryonic hematopoiesis (*30*–*33*).

While the developmental origins of hematopoietic cells and the transcriptional programs involved are well studied, the molecular mechanisms controlling blood formation are less understood. Epigenetic regulation of gene expression involving DNA methylation and demethylation has been implicated largely in adult hematopoiesis (*20*, *34*). Dnmt1, Dnmt3a, Dnmt3b, and Tet enzymes are critical for homeostasis of hematopoietic stem cells (HSCs) in the adult bone marrow. Dnmt1 is essential for HSC self-renewal (*35*, *36*), and Dnmt3a and Dnmt3b are essential for HSC differentiation (*37*, *38*). Tet enzymes are also implicated in adult hematopoiesis and are essential for HSC homeostasis. Loss of Tet1 promotes self-renewal of pro-B cells and causes lymphoma in mice (*39*). *TET2* is frequently mutated in human chronic myelomonocytic leukemia (*40*), and its loss promotes myelodysplastic syndrome (MDS) and myeloid and lymphoid leukemia in mice (*14*, *15*, *41*–*43*). Combined loss of Tet2/3 in adult HSCs blocks differentiation and promotes aggressive leukemias causing rapid death of mice (*44*). Unlike in adult hematopoiesis, roles of Dnmts and Tet enzymes in embryonic hematopoiesis are less investigated. Studies in zebrafish have implicated Dnmt1 and Dnmt3bb.1 in HSPC formation during embryonic development (*45*, *46*). Likewise, Tet2 and Tet3 in zebrafish are implicated in proper activation of Notch signaling for definitive HSPC formation (*47*). In mice, loss of individual Tet genes or combined loss of Tet1 and Tet2 does not impair embryonic hematopoiesis (*7*, *10*, *11*), likely due to potential redundancy among Tet paralogs. Combined loss of all three Tet enzymes blocks gastrulation in mice (*18*) and therefore hinders elucidating the absolute molecular requirements of these enzymes and DNA hydroxylation in epigenetic regulation of mammalian embryonic hematopoiesis. Here, we report that postgastrulation global inducible or endothelial-specific loss of all three Tet enzymes compromises both primitive and definitive embryonic hematopoiesis, leading to mid-gestation lethality. Deficiency of Tet enzymes blocks specification of HSPCs from ECs during embryogenesis in the YS and AGM. At the molecular level, Tet enzymes facilitate hematopoiesis by demethylating promoters, gene bodies, and enhancers in ECs and HSPCs and activating hematopoietic-specific gene expression programs. This involves the expression of nuclear factor κB1 (NFκB1) and hematopoietic transcription factors Gata1, Gata2, Runx1, and Gfi1b. Together, these findings define a critical postgastrulation function for Tet enzymes in mammalian embryonic hematopoiesis and establish a requirement for these enzymes in epigenetic regulation of EC-to-HSC transition during embryogenesis, which is distinct from the requirements of Tet enzymes during adult hematopoiesis where they regulate HSPC maintenance and differentiation (*20*, *39*, *41*, *43*).

## RESULTS

### Inducible postgastrulation global loss of Tet1, Tet2, and Tet3 in mouse embryos leads to mid-gestation embryonic lethality

**Fig. 1.**
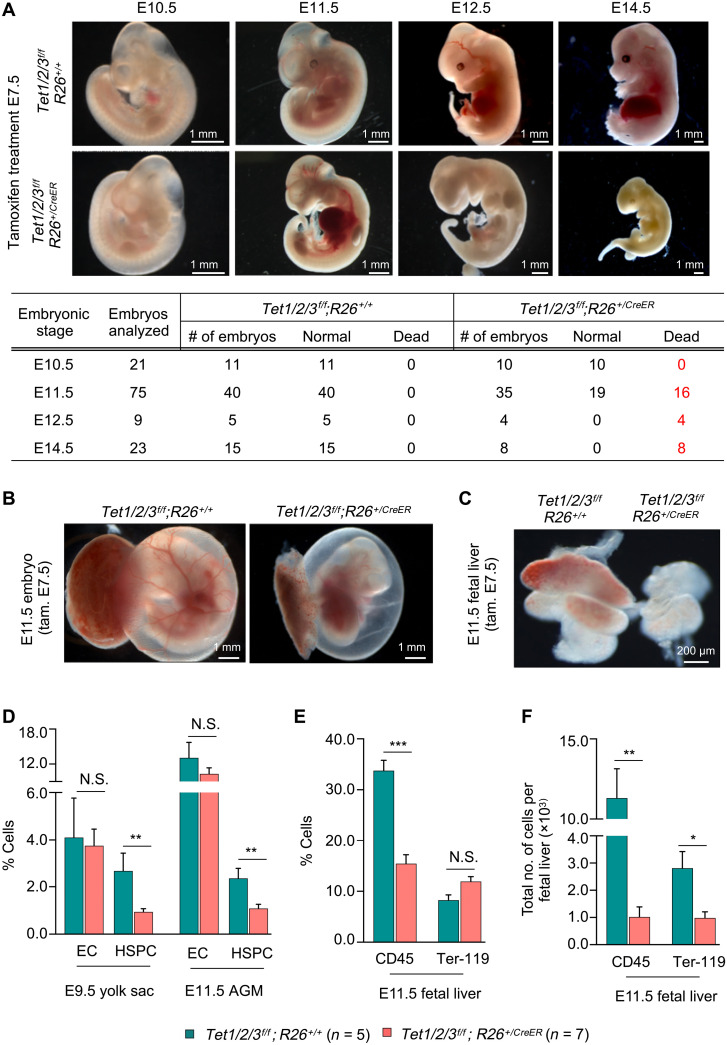
Loss of Tet1/2/3 early in embryogenesis leads to aberrant hematopoiesis. (**A**) Representative gross images of E10.5 to E14.5 embryos of indicated genotypes treated with tamoxifen at E7.5. Total number of normal and dead embryos for each developmental stage is summarized in the table. (**B**) Representative gross images of E11.5 embryos of indicated genotypes attached to yolk sac (YS) and placenta treated with tamoxifen at E7.5. Note the lack of blood in the *Tet1/2/3^f/f^;R26^+/CreER^* YSs. (**C**) Representative gross images of E11.5 embryonic livers of indicated genotypes treated with tamoxifen at E7.5. Note the smaller size and lack of blood in the *Tet1/2/3^f/f^;R26^+/CreER^* fetal liver (FL). (**D**) Quantitation of % HSPCs and % ECs in E9.5 YS and E11.5 AGM of *Tet1/2/3^f/f^;R26^+/CreER^* and littermate control *Tet1/2/3^f/f^;R26^+/+^* embryos by flow cytometry. E9.5 YS HSPCs (Lin^−^CD31^+^c-Kit^+^), ECs (Lin^−^CD31^+^c-Kit^−^), E11.5 AGM HSPCs (CD31^+^c-Kit^+^), and ECs (CD31^+^c-Kit^−^). Lin, lineage. (**E** and **F**) Quantitation of the percent and total number of CD45^+^ hematopoietic cells and Ter-119^+^ erythrocytes in E11.5 FL of *Tet1/2/3^f/f^;R26^+/CreER^* and littermate control *Tet1/2/3^f/f^;R26^+/+^* embryos by flow cytometry. For all panels, data are presented as means ± SEM. Statistically significant (*P < 0.05, **P < 0.01, and ***P < 0.001). N.S. stands for no significant change.

To examine the expression dynamics of Tet enzymes during postgastrulation development, we quantified Tet1, Tet2, and Tet3 mRNA levels in mouse E7.5, E9.5, and E11.5 whole embryo proper. We found that, while Tet1 was expressed at similar levels at all three time points, Tet2 and Tet3 levels were induced at both E9.5 and E11.5 embryos (fig. S1A). Global loss of individual Tet genes in mice is compatible with embryogenesis, partly due to compensatory effects of other Tet genes, but loss of all three Tet genes blocks development at gastrulation (*18*). Therefore, we developed *Tet1*, *Tet2*, and *Tet3* triple floxed *Rosa26^+/CreER^* tamoxifen inducible mice (hereafter referred to as *Tet1/2/3^f/f^;R26^+/CreER^*) to delete all three Tet genes after gastrulation and define their biological requirements in embryogenesis (fig. S1B). We time-mated male *Tet1/2/3^f/f^;R26^+/CreER^* and female *Tet1/2/3^f/f^* mice and administered tamoxifen to pregnant mice at E7.5 to induce deletion of *Tet* genes in the embryos. We confirmed efficient deletion of all three *Tet* genes, complete loss of Tet1/2/3 mRNA, and depletion of 5hmC in *Tet1/2/3^f/f^;R26^+/CreER^* embryos using DNA and RNA isolated from whole embryos (fig. S1, C to E). We analyzed the embryos during mid-gestation from E10.5 to E14.5 (Fig. 1A). At E10.5, *Tet1/2/3^f/f^;R26^+/CreER^* embryos were indistinguishable from their control littermate *Tet1/2/3^f/f^; R26^+/+^* embryos. However, by E11.5, nearly half of *Tet1/2/3^f/f^;R26^+/CreER^* embryos (16 of 35) presented with hemorrhage and died compared to all *Tet1/2/3^f/f^;R26^+/+^* embryos (40 of 40) that developed normally. By E12.5 and E14.5, all *Tet1/2/3^f/f^;R26^+/CreER^* (4 of 4 and 8 of 8) embryos had degenerated and died, while littermate control embryos (5 of 5 and 15 of 15) developed normally. These findings suggest that postgastrulation deletion of Tet enzymes at E7.5 leads to mid-gestation embryonic lethality around E11.5 to E12.5 of development.

### Loss of Tet enzymes leads to reduced number of HSPCs in the YS, AGM, and FL

We noted that, in addition to hemorrhage, E11.5 *Tet1/2/3^f/f^;R26^+/CreER^* embryos had pale YS and smaller FL that visibly lacked blood (Fig. 1, B and C) in the absence of any overt vasculature malformations (fig. S1F). This is reminiscent of a defect in embryonic hematopoiesis potentially involving EC to HSPC specification or aberrant HSPC self-renewal and survival. This prompted us to quantify ECs and HSPCs in sites of primitive (i.e., YS) and definitive (i.e., AGM and FL) hematopoiesis in *Tet1/2/3^f/f^;R26^+/CreER^* and *Tet1/2/3^f/f^;R26^+/+^* littermate control embryos (tamoxifen-treated E7.5). We isolated YS from E9.5 and AGM from E11.5 embryos and quantified ECs (CD31^+^ c-Kit^−^) and HSPCs (CD31^+^ c-Kit^+^) by flow cytometry. While the number of ECs remained unaffected, the number of HSPCs in the YS and AGM of *Tet1/2/3^f/f^;R26^+/CreER^* embryos was reduced to more than half (Fig. 1D). Likewise, both the number and percentage of hematopoietic cells (CD45^+^) in FL were profoundly reduced in *Tet1/2/3^f/f^;R26^+/CreER^* embryos (Fig. 1, E and F). While the number of erythroid cells (Ter-119^+^) in FL was significantly reduced in *Tet1/2/3^f/f^;R26^+/CreER^* embryos, the percentage of erythroid cells was unchanged, owing to the smaller size of the FL of *Tet1/2/3^f/f^;R26^+/CreER^* embryos (Fig. 1, E and F). These findings suggest that Tet-deficient embryos, despite having normal number of ECs at sites of primitive and definitive hematopoiesis, have reduced number of HSPCs and compromised hematopoiesis. To examine whether the reduced number of HSPCs in Tet-deficient embryos is due to increased apoptosis, we quantified the number of apoptotic ECs and HSPCs in wild-type and Tet-deficient AGMs by annexin V and propidium iodide (PI) staining (fig. S1G). While apoptotic ECs were not increased in Tet-deficient AGMs, the apoptotic HPSCs were subtly increased from ~2% in control AGMs to ~3% in Tet-deficient AGMs. This marginal increase in apoptosis cannot account for the profound 50% reduction in HSPC numbers in Tet-deficient AGMs. Next, we analyzed the cell cycle and proliferation of freshly isolated HSPCs and ECs from wild-type and Tet-deficient AGMs by EdU incorporation. We found no differences in the number of cells in each phase of the cell cycle (fig. S1H), suggesting that the reduced number of HSPCs is not due to slow proliferation or delayed cell cycle progression.

### Tet deficiency impairs specification of HSPCs from ECs

**Fig. 2.**
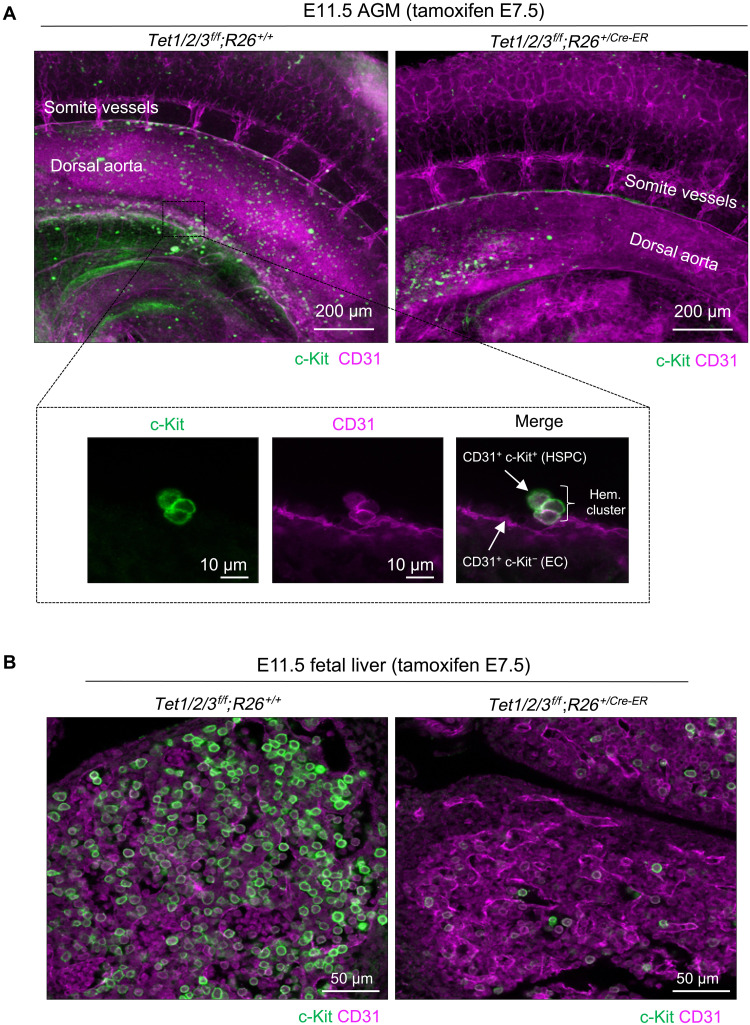
Absence of CD31^+^c-Kit^+^ hematopoietic clusters and floating HSPCs in AGMs of Tet-deficient embryos. (**A**) Whole-mount three-dimensional (3D) images of E11.5 AGM of indicated genotypes stained with CD31 and c-Kit antibodies. CD31^+^ ECs (magenta) and c-Kit^+^ HSPCs (green) within the dorsal aorta region are shown at ×10 magnification. Inset: CD31 and c-Kit double-positive hematopoietic (Hem.) clusters are shown at ×100 magnification. (**B**) Whole-mount 3D images of E11.5 FLs of indicated genotypes stained with CD31 and c-Kit antibodies. CD31^+^ ECs (magenta) and c-Kit^+^ HSPCs (green) are shown at ×40 magnification.

Because HSPCs emerge from ECs during embryogenesis (*23*), we examined whether deficiency of Tet enzymes compromises specification of HSPCs from ECs by visualizing the emergence of hematopoietic clusters from ECs in the dorsal aorta. We isolated the caudal regions of E11.5 *Tet1/2/3^f/f^;R26^+/CreER^* and *Tet1/2/3^f/f^;R26^+/+^* embryos (tamoxifen-treated E7.5) and subjected them to whole mount immunostaining using c-Kit and CD31 antibodies followed by three-dimensional (3D) imaging and reconstruction of the dorsal aorta by confocal microscopy (fig. S2A). We found that the endothelial vasculature (CD31^+^c-Kit^−^) appeared overtly normal and comparable in both wild-type and Tet-deficient embryos (Fig. 2A and fig. S2B). In wild-type embryos, we observed the emergence of CD31^+^c-Kit^+^ hematopoietic clusters from the CD31^+^ c-Kit^−^ endothelial lining of the dorsal aorta along with an abundance of floating c-Kit^+^ HSPCs. However, the presence of hematopoietic clusters and floating HSPCs was profoundly diminished in Tet-deficient AGMs, indicative of impaired specification of HSPCs from ECs (Fig. 2A and fig. S2, B and C). Because HSPCs home into the FL, we also imaged the endothelial and hematopoietic progenitor cells in FL of E11.5 wild-type and Tet-deficient embryos. While CD31^+^ cells appeared overtly normal in Tet-deficient FL, c-Kit^+^ hematopoietic progenitors were profoundly reduced (Fig. 2B). Together, these findings suggest a defect in specification of HSPCs from ECs in Tet-deficient AGMs and confirm a deficit of hematopoietic progenitor cells in sites of embryonic hematopoiesis, in agreement with our flow cytometry data (Fig. 1, D to F).

### Tet-deficient embryonic HSPCs exhibit subtle lineage bias in colony formation assay but form aggressive myeloid malignancies in transplantation assay

**Fig. 3.**
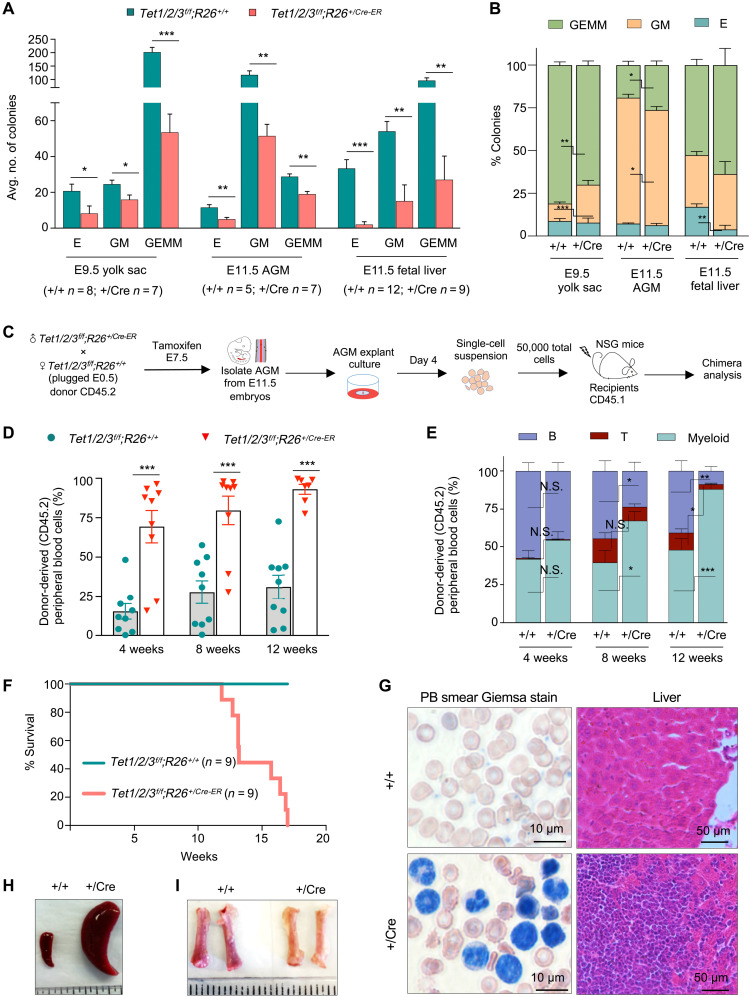
Aberrant colony formation and transplantability of Tet-deficient HSPCs. (**A**) CFU capacity of HSPCs isolated from E9.5 YS, E11.5 AGM, and E11.5 FL of indicated genotypes. E, erythroid (burst-forming unit); GM, granulocyte and macrophage (CFU); GEMM, granulocyte, erythroid, macrophage, and megakaryocyte (CFU). (**B**) Profiles of colonies formed by HSPCs isolated from E9.5 YS, E11.5 AGM, and E11.5 FL. (**C**) Schematic of transplantation assay. (**D**) Donor (CD45.2^+^) chimerism in peripheral blood (PB) of recipient (CD45.1^+^) mice after transplantation. Blood were collected every 4 weeks up to 12 weeks after transplantation. Each dot represents a recipient mouse. (**E**) Lineage distribution of engrafted mice showing CD3 ɛ^+^ T cell (T), B220^+^ B cell (B), and CD11b^+^ myeloid cell percentage in PB within donor-derived (CD45.2^+^) cells every 4 weeks up to 12 weeks after transplantation. (**F**) Kaplan-Meier curve representing percent survival of transplanted NSG mice. (**G**) May-Grünwald-Giemsa–stained PB smears of recipient mice 11 weeks after AGM transplantation shown at ×100 magnification (left) and hematoxylin and eosin staining of livers of recipient mice 12 weeks after AGM transplantation shown at ×40 magnification (right). (**H**) Gross images of the spleen of recipient mice transplanted with AGMs of indicated genotypes at 11 weeks after transplantation. (**I**) Gross images of femurs of recipient mice transplanted with AGMs of indicated genotypes at 11 weeks after transplantation. For all panels, data are presented as means ± SEM. Statistically significant (*P < 0.05, **P < 0.01, and ***P < 0.001).

A key feature of HSPCs is their multipotency and ability to form hematopoietic cell types when cultured in vitro or repopulate the hematopoietic system when transplanted into irradiated adult recipients. To test how loss of Tet enzymes influences the biology of the few embryonic HSPCs that are specified in Tet-deficient AGMs, first, we directly subjected E9.5 YS and E11.5 AGM and FL from wild-type and Tet-deficient embryos to colony-forming unit (CFU) assay. We found that dissociated YS, AGM, and FL from *Tet1/2/3^f/f^;R26^+/CreER^* embryos exhibited lower colony-forming capacity. Tet-deficient YS, AGM, and FL formed significantly fewer erythroid (E), granulocyte and macrophage (GM), as well as granulocyte, erythroid, macrophage, and megakaryocyte (GEMM) colonies compared to their wild-type counterparts (Fig. 3A). This is also consistent with the presence of lower number of HSPCs in *Tet1/2/3^f/f^;R26^+/CreER^* embryos (Figs. 1, D to F, and 2, A and B). For Tet-deficient YS, we observed a significant bias toward forming GM colonies and against forming GEMM colonies. For Tet-deficient AGMs, we found a significant bias toward forming GEMM and against forming GM colonies. There was also a very robust bias against forming E colonies for Tet-deficient FL (Fig. 3B).

Next, we performed a bone marrow transplantation assay. To obtain sufficient cells for transplantation, we cultured AGM explants from E11.5 wild-type and Tet-deficient CD45.2^+^ embryos in defined medium (Fig. 3C). After 4 days, the number of ECs was comparable between wild-type and Tet-deficient AGMs, while the number of HSPCs was fourfold greater in wild-type (20%) than in Tet-deficient (5%) AGMs (fig. S3A). 5hmC was completely depleted in Tet-deficient HSPCs consistent with loss of all three Tet enzymes (fig. S3B). We dissociated the cultured AGMs, pooled them on the basis of their genotypes, and transplanted 50,000 cells (CD45.2^+^) into 2-month-old sublethally irradiated nonobese diabetic (NOD) scid gamma (NSG) (CD45.1^+^) recipient mice (*n* = 9 for each genotype). We examined the presence of donor derived CD45.2^+^ cells in peripheral blood (PB) of recipient mice at 4, 8, and 12 weeks after transplantation. We found that Tet-deficient AGMs, despite having lower number of HSPCs than wild-type AGMs, had a robust engraftment. By 12 weeks, they had reconstituted 85% of PB cells in contrast to 30% reconstitution by wild-type AGMs (Fig. 3D). While all transplanted mice were multilineage, we found a progressively significant expansion of myeloid cells in recipient mice transplanted with Tet-deficient AGMs, with nearly 90% of PB comprising of CD11b^+^ cells in contrast to only 40% in control recipient mice (Fig. 3E). By 12 to 16 weeks, all Tet-deficient AGM transplanted mice succumbed to aggressive myeloid malignancies marked by splenomegaly, infiltration of myeloid cells into the liver, expansion of myeloid cells in PB and bone morrow, and anemia (Fig. 3, F to I, and fig. S3, C to H). Leukemic cells in livers of transplanted mice lacked 5hmC, confirming that they are derived from transplanted Tet-deficient HSPCs (fig. S3I). These phenotypes are consistent with prevalence of myeloid disorders and malignancies linked to Tet2 loss in adult mice (*14*, *41*, *43*) as well as with onset of more robust and aggressive myeloid malignancies associated with inducible combined deletion of *Tet2* and *Tet3* in adult mice (*44*). These findings suggest that Tet-deficient embryonic HSPCs, while exhibiting mild lineage bias in CFU assay, have long-term myeloid differentiation defects when transplanted.

### Endothelial-specific deletion of all three Tet enzymes is sufficient to impair embryonic hematopoiesis and cause mid-gestation embryonic lethality

**Fig. 4.**
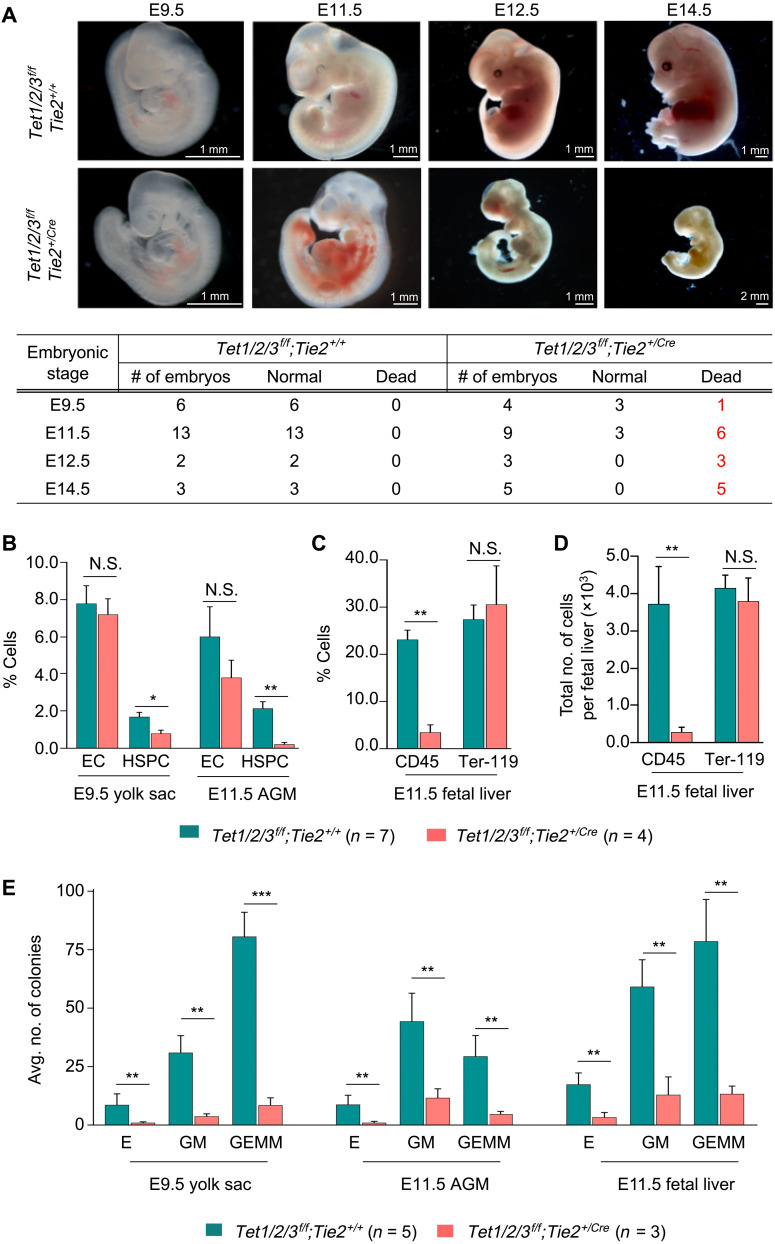
Loss of Tet enzymes in the endothelial lineage leads to impaired hematopoiesis and embryonic lethality. (**A**) Representative gross images of E9.5 to E14.5 embryos of indicated genotypes. Total number of normal and dead embryos for each developmental stage is summarized in the table. (**B**) Quantitation of % HSPCs and %ECs in E9.5 YS and E11.5 AGM of *Tet1/2/3^f/f^;Tie2^+/Cre^* and littermate control *Tet1/2/3^f/f^;Tie2^+/+^* embryos by flow cytometry. (**C** and **D**) Quantitation of percent and total number of CD45^+^ hematopoietic cells and Ter-119^+^ erythrocytes in E11.5 FL of *Tet1/2/3^f/f^;Tie2^+/Cre^* and littermate control *Tet1/2/3^f/f^;Tie2^+/+^* embryos by flow cytometry. (**E**) CFU capacity of hematopoietic progenitors isolated from E9.5 YS, E11.5 AGM, and E11.5 FL of *Tet1/2/3^f/f^;Tie2^+/Cre^* and littermate control embryos. For all panels, data are presented as means ± SEM. Statistically significant (*P < 0.05, **P < 0.01, and ***P < 0.001).

To establish that the hematopoietic defects observed in *Tet1/2/3^f/f^;R26^+/CreER^* embryos are specifically due to loss of Tet enzymes in ECs, we deleted all three Tet genes during embryogenesis using an endothelial-specific *Tie2-Cre* mouse. This strain expresses Cre not only mainly in the ECs but also in the HSCs and weakly in the female germ line. During embryogenesis, it is expressed around E6.5/E7.5 and promotes recombination primarily in the embryonic ECs before the emergence of HSPCs and, therefore, closely mimics tamoxifen-inducible loss of Tet enzymes at E7.5 in our *Tet1/2/3^f/f^;R26^+/CreER^* mouse model. We time-mated *Tet1/2/3^+/f^;Tie2^+/Cre^* males and *Tet1/2/3^f/f^* females and analyzed embryos during mid-gestation from E9.5 to E14.5 (Fig. 4A). We confirmed complete deletion of all three genes by polymerase chain reaction (PCR) using DNA isolated from the FL of *Tet1/2/3^f/f^;Tie2^+/Cre^* embryos (fig. S4, A and B). At E9.5, *Tet1/2/3^f/f^;Tie2^+/Cre^* embryos (3 of 4) were indistinguishable from their control littermate *Tet1/2/3^f/f^;Tie2^+/+^* embryos. However, by E11.5, more than half of *Tet1/2/3^f/f^;Tie2^+/Cre^* embryos (6 of 9) presented with hemorrhage and died compared to all *Tet1/2/3^f/f^;Tie2^+/+^* embryos (13 of 13) that developed normally. By E12.5 and E14.5, all *Tet1/2/3^f/f^;Tie2^+/Cre^* embryos (3 of 3 and 5 of 5) embryos had degenerated and died, while littermate control embryos (2 of 2 and 3 of 3) developed normally (Fig. 4A). These findings suggest that endothelial-specific deletion of Tet enzymes during embryogenesis leads to mid-gestation embryonic lethality around E11.5 to E12.5 of development, which is comparable to the lethality of *Tet1/2/3^f/f^;R26^+/CreER^* embryos that were administered tamoxifen at E7.5 (Fig. 1A).

Next, we quantified ECs (CD31^+^ c-Kit^−^) and HSPCs (CD31^+^ c-Kit^+^) in the AGM of E11.5 and in the YS of E9.5 *Tet1/2/3^f/f^;Tie2^+/Cre^* and *Tet1/2/3^f/f^;Tie2^+/+^* littermate control embryos by flow cytometry (Fig. 4B). While the number of ECs remained unaffected, the number of HSPCs was profoundly reduced in the YS and AGM of *Tet1/2/3^f/f^;Tie2^+/Cre^* embryos. Likewise, the total number and percent of hematopoietic cells (CD45^+^), but not of erythroid cells (Ter-119^+^), were significantly reduced in FL of *Tet1/2/3^f/f^;Tie2^+/Cre^* embryos (Fig. 4, C and D). However, we found that the dissociated YS, AGM, and FL from *Tet1/2/3^f/f^;Tie2^+/Cre^* embryos exhibited lower colony-forming capacity with significantly fewer E, GM, and GEMM colonies compared to their wild-type counterparts (Fig. 4E). These findings suggest that loss of Tet enzymes in the endothelial lineage does not affect EC numbers in the AGM and YS but reduces formation of HSPCs and compromises hematopoiesis. These phenotypes are similar to those observed during inducible loss of Tet enzymes at E7.5 in *Tet1/2/3^f/f^;R26^+/CreER^* embryos (Fig. 1). Therefore, it supports the notion that loss of Tet enzymes in the endothelial lineage is sufficient to impair specification of HSPCs and hematopoiesis.

### Deficiency of Tet enzymes in ECs and HSPCs leads to down-regulation of NFκB1 and master hematopoietic-specific transcription factors

**Fig. 5.**
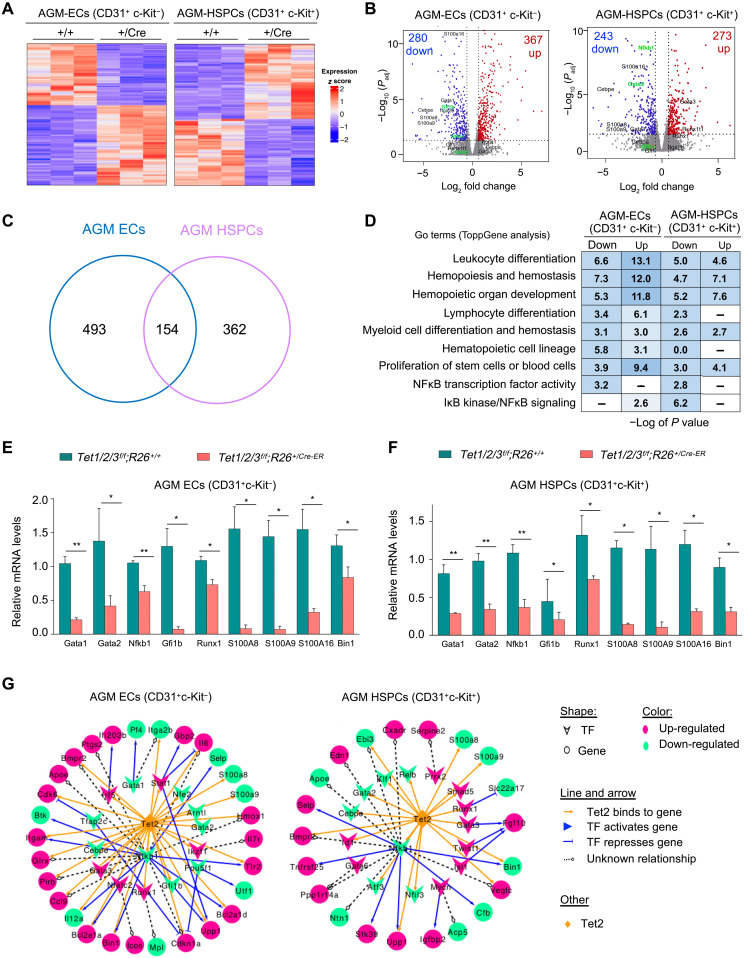
Loss of Tet enzymes in ECs and HSPCs deregulates hematopoiesis gene expression programs. (**A**) Heatmap of expression of differentially expressed genes (DEGs) identified by RNA-seq in ECs and HSPCs isolated from AGMs of E11.5 *Tet1/2/3^f/f^;R26^+/+^* and *Tet1/2/3^f/f^;R26^+/CreER^* embryos that were treated with tamoxifen at E7.5. (**B**) Volcano plots for DEGs identified by RNA-seq (|fold change| > 1.5 and FDR < 0.05). (**C**) Venn diagram showing overlap of DEGs between Tet-deficient ECs and HSPCs. (**D**) Gene Ontology (GO) analysis of up-regulated and down-regulated genes in ECs and HSPCs. “–” in the table indicates GO term not statistically enriched. (**E** and **F**) Quantification of mRNA expression of selected deregulated genes in ECs (E) and HSPCs (F) by RT-qPCR. Data normalized to *Gapdh* expression. (**G**) Gene network analysis of Tet-bound genes in ECs and HSPCs. For (E) and (F), data are presented as means ± SEM. Statistically significant (*P < 0.05, **P < 0.01). TF, transcription factor.

To gain molecular insights into how Tet enzymes regulate specification of HSPCs from ECs during embryogenesis, we analyzed the gene expression program of ECs and HSPCs in the AGM. We isolated RNA from sorted ECs (CD31^+^ c-Kit^−^) and HSPCs (CD31^+^ c-Kit^+^) from E11.5 AGM of *Tet1/2/3^f/f^;R26^+/CreER^* and *Tet1/2/3^f/f^;R26^+/+^* embryos (tamoxifen-treated E7.5) and confirmed loss of all three Tet enzymes by reverse transcription quantitative PCR (RT-qPCR) (fig. S5A) and then subjected them to RNA sequencing (RNA-seq) (three independent batches for each genotype). Principal components analysis revealed distinct clustering of expression profiles of replicates of ECs and HSPCs by each genotype (fig. S5B). We found that, in Tet-deficient AGMs, a comparable number of genes were significantly up- or down-regulated in both ECs (280 down and 367 up) and HSPCs (243 down and 273 up) (Fig. 5, A and B). Of the total number of differentially expressed genes (DEGs), 154 were common between ECs and HSPCs, while 493 were unique to ECs and 362 were unique to HSPCs (Fig. 5C), suggesting that loss of Tet enzymes has both common and distinct effects on the gene expression programs of these cell types. Gene Ontology (GO) analysis of the DEGs revealed enrichment of hematopoiesis terms including HSC lineage commitment, differentiation, and proliferation (Fig. 5D). We found that, among the DEGs in ECs, the NFκB pathway was uniquely enriched in down-regulated genes, while its inhibitor IκB pathway was uniquely enriched in up-regulated genes. Consistently, we found that NFκB1 and several master hematopoietic lineage specifiers including the transcription factors Gata2, Runx1, and Gfi1b as well as the S100A8/9/16 genes were down-regulated in both ECs and HSPCs, as validated by RT-qPCR (Fig. 5, E and F). Expression of maintenance (Dnmt1) and de novo (Dnmt3a and Dnmt3b) DNA methyltransferases was unchanged in both ECs and HSPCs (fig. S5C). These transcription factors, particularly Gata2, Runx1, and Gfi1b, have been implicated in specification of HSPCs from ECs and are critical for embryonic hematopoiesis (*32*–*35*). To gain insights into the hierarchical regulation of these factors during hematopoiesis, we built a transcriptional regulatory network. We classified the DEGs as transcription factors and nontranscription factor genes and established a regulatory relationship among them on the basis of a transcriptional regulatory relationship unraveled by sentence-based text-mining (TRRUST) database. This analysis placed Tet enzymes and NFκB1 at the center of a regulatory network upstream of Gata2, Runx1, Gfi1b, and other regulators of hematopoiesis (Fig. 5G). Because genomic occupancies of Tet enzymes in the embryonic ECs or HSPCs have not been mapped yet, we used the available Tet ChIP-seq data in the bone marrow multipotent progenitors (MPPs) and ESCs (*48*, *49*) to examine whether Tet enzymes are enriched at these genes. We found a robust enrichment of Tet1 and Tet2 at promoter and gene bodies of NFκB1, Gata2, Runx1, and Gfi1b, suggesting that they are direct targets of Tet enzymes (fig. S5D). Together, these findings suggest that Tet enzymes are essential for hematopoiesis gene expression programs in ECs and HSPCs by regulating proper expression of NFκB1 and the master hematopoietic lineage specifiers Gata2, Runx1, and Gfi1b. Of note, these factors were also down-regulated during spontaneous differentiation of Tet1/2/3 TKO ESCs to embryoid bodies (EBs) that failed to form hematopoietic cells (fig. S5, E and F), further underscoring the significance of Tet enzymes in regulation of NFκB1, hematopoietic-specific transcription factors, and hematopoiesis.

### Loss of Tet enzymes in ECs and HSPCs leads to DNA hypermethylation across promoters, enhancers, and gene bodies of hematopoiesis genes

**Fig. 6.**
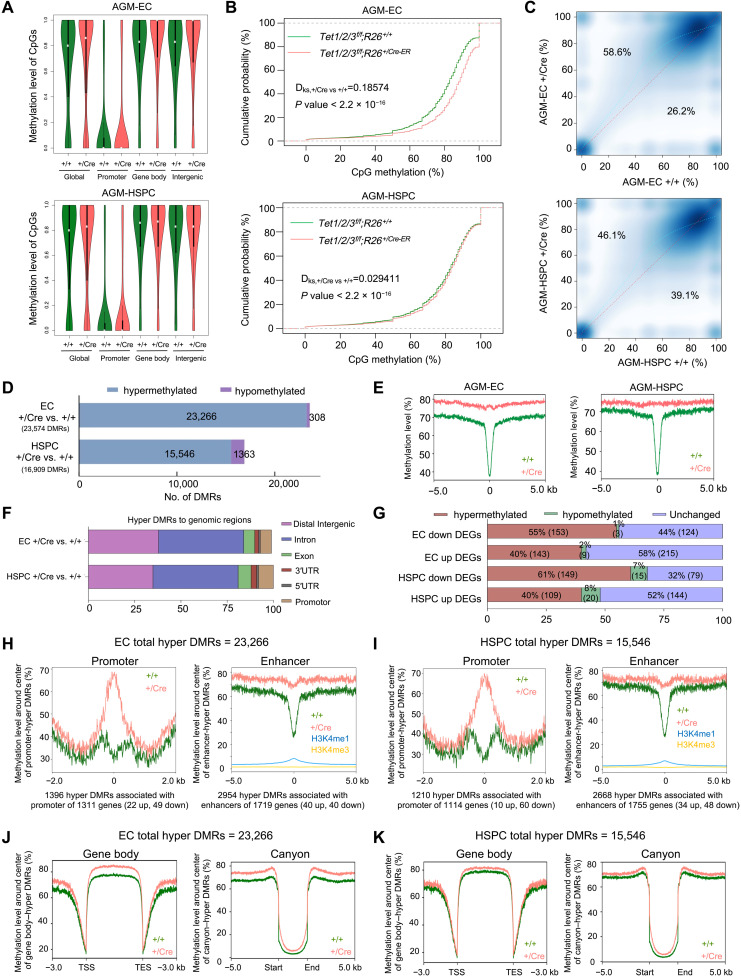
Global and hematopoiesis gene-specific DNA hypermethylation in Tet-deficient ECs and HSPCs. (**A**) Violin plots showing CpG methylation levels in ECs and HSPCs isolated from AGMs of E11.5 *Tet1/2/3^f/f^;R26^+/+^* and *Tet1/2/3^f/f^;R26^+/CreER^* embryos that were treated with tamoxifen at E7.5. (**B**) Cumulative plot of average DNA methylation value within 1-kb windows across the mouse genome in ECs and HSPCs of indicated genotypes. One-kilobase windows with three or more CpGs were considered for the analysis. Kolmogorov-Smirnov test was used to calculate *D* statistics and *P* value to examine whether methylation levels were significantly different. (**C**) Density plot of average DNA methylation values within 1-kb windows across the genome. 5-methylcytosine (5mC) values in wild-type samples (*x* axis) were compared to +/Cre samples (y axis). Locally estimated scatterplot smoothing (LOESS) regression (light blue dashed line) is displayed. (**D**) Total numbers of hypermethylated and hypo-methylated DMRs in ECs and HSPCs. DMRs are regions with more than three DMSs with a maximum distance of 300 bp, having a methylation difference ≥ 20% and FDR < 0.01. (**E**) Average 5mC levels at ±5 kb of all DMR centers in ECs and HSPCs. (**F**) Assignment of hypermethylated DMRs to genomic regions. 3′UTR, 3′ untranslated region. (**G**) Total numbers and percentages of hypermethylated and hypomethylated DEGs. (**H** to **K**) Average 5mC levels around the promoters (±2 kb of TSSs), enhancers (H3K4me1 high and H3K4me3 low, ±5 kb), gene bodies (±3 kb), and DNA methylation canyons (±5 kb) in ECs and HSPCs. Total number of hypermethylated DMRs (hyper DMRs) and the total number of DEGs overlapping with hyper DMRs at promoters and enhancers are displayed.

**Fig. 7.**
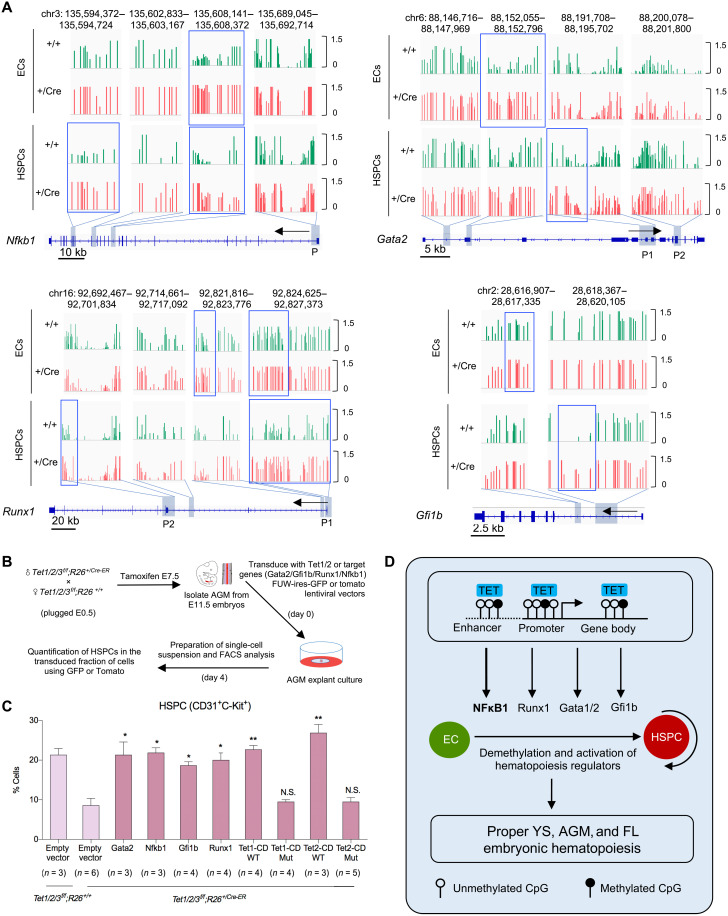
NFκB1 and master hematopoietic transcription factors are hypermethylated in Tet-deficient ECs and HSPCs and their reexpression or that of Tet catalytic domain ameliorates hematopoiesis defects in Tet-deficient AGMs. (**A**) Genome browser tracks showing 5mC levels at selected down-regulated master hematopoiesis transcription factors. Each vertical line on tacks represents a 5mC site. Blue frames indicate regions with more robust differences in methylation. P stands for promoter. (**B**) Schematic of experimental design to test whether reexpression of Tet1/2 catalytic domains or NFκB1, Gata2, Gfi1b, and Runx1 can rescue hematopoietic defects in Tet-deficient AGMs. (**C**) Quantification of % HSPCs (CD31^+^c-Kit^+^) in AGMs of indicated genotypes by flow cytometry. (**D**) Model depicting the role of Tet enzymes in epigenetic regulation of embryonic hematopoiesis involving a Tet-NFκB1–(Gata2, Runx1, and Gfi1b) axis. For all panels, data are presented as means ± SEM. Statistically significant (*P < 0.05 and **P < 0.01).

Because Tet enzymes promote DNA demethylation, we examined how their loss affects the global and gene-specific DNA methylation dynamics of ECs and HPSCs, and whether that influences the hematopoiesis gene expression programs including the regulation of hematopoietic lineage specifiers. To this end, we isolated DNA from sorted ECs (CD31^+^ c-Kit^−^) and HSPCs (CD31^+^ c-Kit^+^) from E11.5 AGM of *Tet1/2/3^f/f^;R26^+/CreER^* and *Tet1/2/3^f/f^;R26^+/+^* embryos (tamoxifen-treated E7.5) and subjected them to whole-genome bisulfite sequencing (WGBS). We found that, while global DNA methylation was increased in both Tet-deficient ECs and HSPCs compared to their respective wild-type counterparts, the increase was more pronounced in ECs. Percent methylated CpGs was elevated by ~8% in ECs and ~2.5% in HSPCs (fig. S6A). In ECs, this increase was prominently seen at gene bodies and intergenic regions (Fig. 6A). Likewise, cumulative distribution (Fig. 6B) and density (Fig. 6C) of average DNA methylation across 1-kb windows of the genome showed a significant shift to increased methylation in both Tet-deficient ECs and HSPCs, with the increase being more robust in ECs than in HSPCs. Next, we identified differentially methylated regions (DMRs) in Tet-deficient ECs and HSPCs versus their wild-type counterparts. We defined DMRs as regions with a methylation difference of at least 20%, containing at least three differentially methylated sites (DMS) separated by a maximum distance of 300 base pairs (bp). Increased DNA methylation was observed in both Tet-deficient ECs and HSPCs (Fig. 6, A to C). We found a higher number of DMRs in ECs (23,574) than in HSPCs (16,909). The vast majority of DMRs were hypermethylated in ECs (23,266 hyper versus 308 hypo) and in HSPCs (15,546 hyper versus 1363 hypo) (Fig. 6D). The methylation difference was pronounced at the center and along the ±5 kb of the DMR (Fig. 6E), with most of the DMRs being 20 to 50% hypermethylated (fig. S6B). The hypermethylated DMRs were largely mapped to introns and distal intergenic regions followed by promoters and exons (Fig. 6F) and were associated with ~8000 genes in ECs and ~7000 genes in HSPCs (fig. S6C). Overall, 55 and 61% of down-regulated genes in ECs and HSPCs, respectively, contained hypermethylated DMRs (Fig. 6G). We found that 6% (1396 of 23,266) of hypermethylated DMRs in ECs were at promoters and associated with 1311 genes (49 down-regulated in Tet-deficient ECs) and 13% (2954 of 23,266) of hypermethylated DMRs were at enhancers and associated with 1719 genes (40 down-regulated in Tet-deficient ECs) (Fig. 6H). A similar trend was observed in HSPCs where we found 8% (1210 of 15,546) hypermethylated DMRs at promoters associated with 1114 genes (60 down-regulated in Tet-deficient ECs) and 17% (2668 of 15,546) of hypermethylated DMRs were at enhancers and associated with 1755 genes (48 down-regulated in HSPCs) (Fig. 6I). DNA methylation was also increased across gene bodies and at DNA methylation canyons in both ECs and HPSCs (Fig. 6, J and K). GO analysis of DEGs associated with promoter and enhancer DMRs in ECs (fig. S6D) and of the down-regulated DEGs associated with hypermethylated DMRs in ECs and HSPCs (fig. S6E) revealed enrichment of hematopoiesis terms and processes and included NFκB1, Gata2, Gfi1b, Runx1, Cebpe, and several other regulators of hematopoiesis. DNA methylation levels were elevated at their regulatory regions in both Tet-deficient ECs and HSPCs, with the increase more notable in ECs (Fig. 7A and fig. S7A). *Nfκb1* had increased methylation at the vicinity of its transcription start site (TSS) and across its gene body. *Runx1* had increased methylation near its known promoters and several regions in its gene body. Likewise, DNA methylation was robustly increased at *Gata2* within its gene body. Both *Gfi1b* and *Cebpe* had modest increases in DNA methylation at and around their TSSs, while *Bin2* had a robust increase in methylation across its gene body. Together, these findings suggest that loss of Tet enzymes in ECs and HSPCs leads to global and gene-specific hypermethylation involving *Nfκb1* and other master transcription factors essential for hematopoiesis that are down-regulated in Tet-deficient ECs and HSPCs.

### Reexpression of Tet catalytic domain or Tet downstream effectors (NFκB1, Gata2, Gfi1b, and Runx1) ameliorates the impaired hematopoiesis in Tet-deficient AGMs

Because loss of Tet enzymes leads to DNA hypermethylation and silencing of their effector hematopoietic lineage specifier genes, we examined whether reexpression of Tet catalytic domain or their downstream targets (NFκB1, Gfi1b, Gata2, and Runx1) can rescue hematopoiesis defects of Tet-deficient AGMs in an explant culture system. We isolated AGMs from E11.5 *Tet1/2/3^f/f^;R26^+/CreER^* and *Tet1/2/3^f/f^;R26^+/+^* embryos (tamoxifen-treated E7.5); transduced them with tdTomato or green fluorescent protein (GFP) lentiviral vectors expressing the Tet1 or Tet2 catalytic domain (Tet1 CD or Tet2 CD), their mutant forms (Tet1 CD-Mut or Tet2 CD-Mut), NFκB1, Gata2, Gfi1b, Runx1, or an empty vector; and cultured them in defined medium for 4 days (Fig. 7B). We quantified the number of HSPCs (CD31^+^ c-Kit^+^) by flow cytometry in tdTomato^+^ or GFP^+^ fraction of cells of each AGM (Fig. 7C). We found that Tet-deficient AGMs transduced with Tet1CD or Tet2CD had significantly more HSPCs (~22 and ~26%, respectively) in contrast to Tet-deficient AGMs transduced with their mutant forms or an empty vector, which had only ~10% HSPCs. Likewise, Tet-deficient AGMs transduced with NFκB1 or Gata2 or Gfi1b or Runx1 had significantly more HSPCs (~22, ~21, ~19, and ~ 20%, respectively) compared to ~10% HSPCs in Tet-deficient AGMs expressing an empty vector. The % HSPCs in Tet-deficient AGMs expressing Tet1CD, Tet2CD, NFκB1, Gata2, Gfi1b, or Runx1 was comparable to the % HSPCs in wild-type AGMs expressing an empty vector. Of note, we did not observe any significant increase in the number of ECs due to expression of Tet domains or any of the transcription factors (fig. S7B). We also found that reexpression of Tet1CD or Tet2CD restored or improved expression of NFκB1 and other hematopoietic transcription factors in Tet-deficient ECs and HSPCs (fig. S7C). Reexpression of NFκB1 or Gata2 or Runx1 improved expression of some hematopoiesis transcription factors (fig. S7C). These findings suggest that the catalytic activity of Tet enzymes is essential for specification and expansion of HSPCs in the AGM and that the catalytic domain of any Tet or any of their downstream hematopoietic transcription factors or NFκB1 is sufficient to restore proper hematopoiesis in Tet-deficient AGMs to near normal levels.

We then assessed whether or not reexpression of Tet enzymes or any of their downstream effectors can correct colony formation defects of Tet-deficient AGMs. To this end, we transduced Tet-deficient AGMs with Tet1CD or Tet2 CD or NFκB1 or Gfi1b or Gata2 or Runx1 or an empty vector and subjected them to CFU and serial replating assays. We noted that, in contrast to freshly isolated Tet-deficient AGMs that have reduced colony forming potential (Fig. 3A), cultured Tet-deficient AGMs (for 1 week to allow for lentiviral transductions and expansion) had increased colony forming and replating capacity (fig. S7D). This suggests that Tet deficiency in the cultured AGMs (as was in transplanted AGMs) promotes HSPC transformation. Nevertheless, overexpression of Tet1CD or Tet2CD or any of the transcription factors robustly corrected the increased serial replating potential of Tet-deficient HSPCs (fig. S7D). Together, these rescue experiments functionally validate the involvement of Tet enzymes, their enzymatic activity, and downstream effectors in specification of HSPCs from ECs during embryonic hematopoiesis and in the biology of HSPCs (Fig. 7D).

## DISCUSSION

Germline deletion of all three Tet genes in mice blocks development at gastrulation (*18*). Our study examines the developmental consequences of combined loss of all three Tet enzymes in postgastrulation embryonic development. Here, we provide five lines of evidence in support of an essential function for Tet enzymes in specification of HSPCs from ECs during embryonic hematopoiesis. (i) Inducible global deletion of all three Tet enzymes at E7.5, or their endothelial-specific loss alone, severely reduced the number of hematopoietic progenitors in the E9.5 YS and E11.5 AGM and FL and led to embryonic lethality. (ii) Deficiency of Tet enzymes impaired specification of HSPCs from ECs. (iii) Loss of Tet enzymes in ECs and HSPCs led to global and hematopoiesis gene-specific DNA hypermethylation. (iv) NFκB1, Gata2, Runx1, and Gfi1b are direct downstream targets of Tet enzymes in ECs and HSPCs, and loss of Tet enzymes led to their promoter, gene body, and enhancer hypermethylation and subsequent down-regulation. (v) Reexpression of Tet catalytic domain or Tet downstream effectors (NFκB1, Gata2, Gfi1b, and Runx1) ameliorated the impaired hematopoiesis in Tet-deficient AGMs. These findings establish Tet enzymes as essential regulators of embryonic hematopoiesis where they promote demethylation and expression of NFκB1 and master hematopoietic lineage specifiers to facilitate endothelial to hematopoietic transition. This is distinct from the requirements of Tet enzymes during adult hematopoiesis where they regulate HSPC self-renewal and differentiation (*14*, *39*, *41*, *43*, *44*).

Both the global deletion of Tet enzymes at E7.5 and their endothelial-specific deletion by Tie2-Cre led to notably similar hematopoiesis defects and lethality at ~E11.5. This suggests that loss of Tet enzymes in the ECs alone is sufficient to impair embryonic hematopoiesis and cause lethality. However, it is possible that Tet deficiency leads to defects in nonhematopoietic cell types that have endothelial origins and that lethality may not be entirely due to hematopoietic defects. For example, heart development has endothelial origins, and Nkx2.5-Cre–mediated combined deletion of Tet2 and Tet3 in the cardiac lineage leads to cardiomyopathy with embryos not surviving beyond E16.5 (*19*). Nonetheless, phenotypically Tet1/2/3-deficient embryos die earlier than Tet2/3 Nkx2.5-Cre embryos (E11.5 versus E16.5) and with more severe defects (excessive hemorrhage and complete degeneration versus reduced ventricular wall thickness). This supports an early embryonic hematopoiesis defect as a more likely cause of lethality of Tet1/2/3-deficient embryos.

The Rosa26-CreER and Tie2-Cre models efficiently deleted Tet enzymes and depleted 5hmC early enough in embryogenesis to allow for examining the impact of Tet and 5hmC loss on primitive and definitive hematopoiesis in the YS and AGM, respectively. We note that lethality is slightly earlier and more severe in Tie2-Cre model than in Rosa26-CreER model. This could be due to subtle differences in time frame of recombination between the two strains (Tie2-Cre E6.5/E7.5 versus Rosa26-CreER tamoxifen treatment at E7.5 and requiring a few hours to be taken up by cells). Our findings that hematopoietic progenitor numbers are markedly reduced in the YS and AGM, in the absence of any significant changes in apoptosis or proliferation, point to a defect in the endothelial compartment from where HSPCs are derived. However, Tet deficiency neither affected the number of CD31^+^ c-Kit^−^ ECs nor the gross and histological features of the YS and arterial endothelium. Instead, Tet-deficient ECs failed to undergo endothelial-to-hematopoietic transition and could not specify CD31^+^ c-Kit^+^ hematopoietic clusters and HSPCs. This is captured by the absence of hematopoietic clusters and floating HSPCs in the dorsal aorta of Tet-deficient embryos. This defect is reminiscent of impaired hematopoiesis and embryonic lethality observed in knockout models of key hematopoietic-specific transcription factors such as Runx1 and Gata2 (*30*, *31*), where EC number and arterial vasculature remain largely unaffected, but ECs fail to specify HSPCs. We find that Runx1 and Gata2 and several other key hematopoiesis-specific transcription factors are significantly down-regulated in Tet-deficient ECs and HSPCs. Their reexpression in Tet-deficient AGMs corrects aberrant colony formation and hematopoiesis defects. This supports that Runx1 and Gata2 are some of the main targets of Tet enzymes through which Tets regulate hematopoiesis. This may also explain why Tet1/2/3-deficient embryos appear more profoundly affected than embryos lacking Runx1 or Gata2 alone. Direct comparison of transcriptomes of Tet-deficient embryonic ECs/HSPCs with those of Runx1- or Gata2-deficient embryonic ECs/HSPCs may elaborate more on parallel or distinct downstream gene expression changes responsible for phenotypes of *Tet*, *Runx1*, and *Gata2* null embryos.

Our findings also establish that Tet deficiency affects the biology of embryonic HSPCs. The few HSPCs present in Tet-deficient AGMs do not express Tet1/2/3 mRNAs and therefore did not arise from ECs with possible partial deletion of Tet genes. Their reduced multipotency in CFU assay suggest that they have compromised differentiation with mild lineage bias, which is consistent with their reduced expression of hematopoietic lineage regulators. HSPCs derived from cultured Tet-deficient AGMs showed increased CFU and replating potential and, when transplanted, formed very aggressive myeloid leukemia with complete penetrance and latency of ~12 to16 weeks. This is very similar to the aggressive myeloid leukemia reported in mice with inducible deletion of *Tet2* and *Tet3* in adult HSPCs that also have complete penetrance but a much shorter latency of 3 to 7 weeks (*44*) or to the phenotypes seen in Tet2-deficient mice that develop MDS and leukemia with 50% penetrance by 1 year of age (*14*, *43*). The disease pathology is different from that of *Tet1* knockout or *Tet1/2* double-knockout mice that develop B cell malignancies at ~18 to 24 months of age (*39*, *50*). These differences in embryonic HSPCs versus cultured or transplanted HSPCs are likely due to (i) the short duration of embryogenesis or CFU assay not being enough for transformation of HSPCs or (ii) differences in embryonic FL versus adult bone marrow microenvironment. Deleting Tet enzymes in HSPCs after E11.5 using a Vav-Cre model may dissect if Tet-deficient embryonic HSPCs encounter differentiation defects, lineage bias, and transformation later during embryogenesis.

While a common set of hematopoietic transcription factors and NFκB1 are deregulated in ECs and HSPCs, their influence on hematopoiesis is different. This is evident from the deregulation of distinct sets of 493 and 362 genes in ECs and HSPCs, respectively, as well as their impact on the biology of ECs (failure to specify to HSPCs) and in properties of HSPCs (failure to differentiate properly). Our findings that Tet enzymes regulate NFκB1 and master hematopoietic transcription factors in ECs and HSPCs by demethylating them provide mechanistic insights into proper activation of hematopoiesis programs during embryogenesis. Hierarchical clustering of deregulated transcription factors in Tet-deficient ECs and HSPCs places Tet enzymes upstream of NFκB1 and several master regulators of hematopoiesis including Gata2, Runx1, and Gfi1b. Consistently, loss of Tet enzymes leads to their promoter, enhancer, and gene body hypermethylation and subsequent down-regulation. NFκB1 expression rescued aberrant hematopoiesis in Tet-deficient AGMs, which implies that NFκB1 works upstream of hematopoietic regulators to regulate embryonic hematopoiesis. NFκB1 down-regulation in Tet-deficient cells is concomitant with dysregulation of selected inflammatory genes, which may also influence HSPC biology. Genomic distribution of Tet enzymes in embryonic ECs and HSPCs has not been mapped. However, in other progenitor cell types, it has been shown that Tet1 is largely associated with promoters and Tet2 with enhancers (*48*, *49*). Although our data support compensatory roles among Tet enzymes in activation of hematopoietic programs, there could be specific requirements for each Tet enzyme as is observed in adult HSPCs (*11*, *39*, *41*–*44*). A comprehensive mapping of genomic occupancy of all three Tet proteins in embryonic ECs and HSPCs and integrating it with expression and methylation changes at gene regulatory regions is needed to elaborate more accurately on any overlapping or unique functions for the three Tet enzymes in embryonic hematopoiesis. A recent study has identified a large set of active enhancers in embryonic arterial endothelium and HSPCs (*51*). Given the prevalence of Tet enzymes at enhancers, it will be of interest to examine how their loss influences priming and activation of these enhancers during embryonic hematopoiesis. Tet enzymes can regulate gene expression not only by its canonical roles in DNA demethylation but also by its noncanonical roles in formation of chromatin activating and repressive complexes (*2*). Although the catalytic activity of Tets was sufficient to ameliorate hematopoiesis defects of Tet-deficient AGMs, it is possible that Tet enzymes can also regulate gene expression programs by noncatalytic means in embryonic ECs and HSPCs, as it is shown for Tet2 in adult HSPCs (*43*).

In summary, mammalian embryonic hematopoiesis is a critical postgastrulation event that entails specification of hematopoietic progenitor cells from ECs first in the YS (primitive hematopoiesis) and later in the arterial vasculature (definitive hematopoiesis). This involves an endothelial-to-hematopoietic transition that is tightly regulated by defined transcriptional programs but poorly understood epigenetic mechanisms. We identify Tet enzymes as prime regulators of activation of hematopoietic programs in ECs for specification and emergence of HSPCs during embryogenesis by promoting demethylation and timely expression of master hematopoiesis transcription factors. An essential role for Tet enzymes in embryonic hematopoiesis appears to be conserved between mammals and lower vertebrates. In zebrafish, Tet2 regulates differentiation of embryonic erythroid precursors by demethylating and activating Gata1 (*52*), and combined loss of Tet2 and Tet3 affects differentiation of ECs to HECs due to failed activation of Notch1-Gata2 signaling (*47*). These studies do not account for Tet1, which is not induced during zebrafish development. Although we do not find perturbation of Notch1 in Tet-deficient mouse ECs, our data support a conserved role for Tet proteins in vertebrate embryonic hematopoiesis, albeit involving different signaling pathways but some common downstream master transcription factors, including Gata1 and Gata2. Our findings broaden our knowledge of epigenetic regulation of mammalian hematopoiesis and have implications in understanding the molecular basis of normal and abnormal hematopoiesis during development. It could also enhance efforts to generate blood cells from pluripotent stem cells, especially because we find that Tet deficiency blocks formation of hematopoietic cells during differentiation of ESCs to EBs, which is coupled with poor expression of key hematopoietic-specific transcription factors.

## MATERIALS AND METHODS

### Generation of Tet1/2/3 R26-Cre-ER and Tet1/2/3 Tie2-Cre mice

All mouse studies were conducted in a specific pathogen–free barrier facility in accordance with our Institutional Animal Care And Use Committee (IACUC)–approved protocols overseen by the Institute for Animal Studies at Albert Einstein College of Medicine. Homozygous *Tet1^f/f^*, *Tet2^f/f^* (*50*), and *Tet3^f/f^* (*17*) mice were intercrossed to generate homozygous triple conditional *Tet1/2/3^f/f^* mice, which were then crossed to *Rosa26-Cre^ER^* mice [the Jackson Labortory (Jax lab), stock no. 004847] to generate *Tet1/2/3^f/f^;R26^+/CreER^* mice. Male *Tet1/2/3^f/f^;R26^+/CreER^* mice were bred to female *Tet1/2/3^f/f^* mice to obtain littermate *Tet1/2/3^f/f^;R26^+/CreER^* and *Tet1/2/3^f/f^;R26^+/+^* embryos for use in experiments. *Tet1/2/3^f/f^* mice were crossed to *Tie2-Cre* mice (Jax lab, stock no. 004128) to generate *Tet1/2/3^+/f^;Tie2^+/Cre^* mice. Male *Tet1/2/3^+/f^;Tie2^+/Cre^* mice were bred to female *Tet1/2/3^f/f^* mice to obtain littermate *Tet1/2/3^f/f^;Tie2^+/Cre^* and *Tet1/2/3^f/f^;Tie2^+/+^* embryos for use in experiments. For genotyping of mice, DNA was extracted from a tail tip biopsy following the standard protocols using proteinase K (Promega, V3021). PCR reactions were carried out using primers listed in table S1.

### Tamoxifen treatment, isolation of YS/FL/AGM, and explant cultures

Tamoxifen (Sigma-Aldrich, T5648-5g) was dissolved in corn oil (Sigma-Aldrich, C8367) and injected into timed-mated pregnant mice (intraperitoneal, 80 mg/kg of body weight) at E7.5. Embryos were isolated from the uteri under a dissecting microscope. Embryos were transferred to cold Dulbecco’s phosphate-buffered saline (DPBS) (Corning, 21-031-CV) for imaging and harvesting YS, FL, and AGM. AGM isolation and establishment of explant cultures were performed as described before (*32*). For genotyping embryos, DNA was extracted from a small piece of head with the Fast KAPA Mouse Genotyping Kit (KAPA Biosystems, KK7352), and PCR reactions were carried out according to the manufacturer’s instructions using primers listed in table S1.

### Lentiviral preparation and transduction of cultured AGM

Transgenes of hemagglutinin (HA)–tagged wild-type and mutant mouse Tet1 and Tet2 catalytic domains, NFκB1 and Gfi1b, were cloned into FUW-HA-2A-tdTomato vector. HA-tagged mouse Gata2 transgene was cloned into FUW-HA-IRES-GFP vector. HA-tagged mouse Runx1 transgene was cloned into MSCV-Runx1-HA-IRES-GFP vector. Lenti and retro viruses were prepared as described before (*32*, *43*) and concentrated using Lenti-X concentrator (Clontech Laboratories, 631231). E11.5 AGMs were isolated from *Tet1/2/3^f/f^;R26^+/CreER^* and their littermate control *Tet1/2/3^f/f^;R26^+/+^* embryos and transduced with 30 μl of concentrated lentivirus (1:100 concentration) and 10 μl of Lentiblast Premium (OZ Biosciences, LBPX500) in 300 μl of cultured medium, centrifuged at 900 rpm for 90 min at room temperature, and cultured for 4 days. The AGMs were treated with 0.1% collagenase (Gibco, 17018-029) with 10% fetal bovine serum (FBS) at 37°C for 30 min and disassociated by passing through a 27-gauge needle. Dissociated cells were incubated first with Purified anti-mouse CD16/32 Antibody (Fc Block) (BioLegend, 101302) and then with allophycocyanin (APC)–conjugated c-kit antibody and phycoerythrin (PE)/fluorescein isothiocyanate (FITC)/PE–Cyanine7 (Cy7)–conjugated CD31 antibody and analyzed by BD LSR II Yellow. CD31^+^c-kit^−^ ECs and CD31^+^c-kit^+^ HSPCs were gated from all GFP^+^ or tdTomato^+^ cells.

### Colony formation assay

Colony formation and serial replating assays were performed following the standard methods (*32*, *43*). Briefly, isolated E11.5 AGMs or E11.5 FLs or E9.5 YSs or E11.5 AGMs that were transduced with different vectors were dissociated into single cells using a 27-gauge needle in Iscove’s modified Dulbecco’s media (IMDM). For the transduced AGMs, tdTomato^+^ or GFP^+^ cells were sorted by flow cytometry. A total of 10,000 AGM cells or 4000 tdTomato^+^ or GFP^+^ cells from transduced AGMs were suspended in 300 μl of IMDM medium with 20% FBS and 10% penicillin/streptomycin. This 300 μl of cell suspension was mixed with 3 ml of MethoCult GF (STEMCELL Technologies, M03434) and cultured in two 35-mm dishes at 37°C with 5% CO_2_ for 7 days. Colonies were counted under a microscope. For the serial replating assay, the total CFU cells were washed by PBS, and 2000 cells were cultured in one 35-mm dishes with two replicates. A total of four rounds of replating was performed. For immunostaining of day 7 CFU cells, cells were seeded on coverslips coated with RetroNectin (Takara, T100A) for 6 hours. Cells were fixed with prechilled methanol for 5 min, 2% paraformaldehyde for 10 min, and permeabilized with 0.1% Triton X-100 for 15 min and blocked [2% bovine serum albumin (BSA), 5% donkey serum, 0.1% Triton X-100 in PBS]. For 5hmC staining, cells were treated with 4 N HCl for 20 min and neutralized by 10 mM tris for 10 min and then blocked. Cells were incubated with primary antibodies (Tet1, 1:500; EpiGentek, A-1020-100; Tet2,1:500; Abcam, ab124297; 5hmC, 1:1000; Active Motif, 39769) overnight at 4°C. Cells were washed, stained with Alexa Fluor 594–anti-rabbit antibody and 4′,6-diamidino-2-phenylindole (DAPI), and imaged using an inverted Zeiss fluorescence microscope.

### Transplantation

Transplantation studies were conducted following previously described protocols (*32*, *53*) in accordance with our IACUC-approved protocols overseen by the Institute for Animal Studies at Albert Einstein College of Medicine. Briefly, 8- to 10-week-old male NOD-*scid* IL2Rgamma^null^ (NSG) CD45.1^+^ mice (bred in house or purchased from Jax lab, stock no. 005557) were sublethally irradiated (2.0 gray) 24 hours before transplantation. A total of 50,000 nucleated cells from E11.5 AGM explants (CD45.2^+^) were suspended in 200 μl of RPMI1640 medium (Corning, 31918013) and retro-orbitally injected into CD45.1^+^ NSG mice. Transplanted mice were given sulfamethoxazole and trimethoprim (Ani, NDC 62559-550-16) in drinking water to prevent infections. Mice were bled every 4 weeks (for 16 weeks) to monitor donor contribution. For PB analysis, mice were bled retro-orbitally, and 100 μl of blood was collected in 0.5-μm EDTA, treated with red blood cell lysis buffer and protein extraction buffer (PEB) buffer solution, and then stained with antibodies for flow cytometry analysis to quantify CD45.2^+^ cells and donor contribution. Blood from 11-week post-transplantation mice was smeared for May-Grünwald-Giemsa staining as previously described (*44*). Moribund transplanted mice were euthanized, and spleens and livers were harvested, imaged, weighed, and fixed in 10% formalin solution at 4°C.Tissues were blocked, sectioned, and subjected to staining with hematoxylin and eosin, anti-5hmC antibody (1:1000; Active Motif, 39769), or anti-myeloperoxidase antibody (1:400; Thermo Fisher Scientific, RB-373-A1) following the standard protocols at Einstein Histopathology Core. Slides were imaged with an inverted Zeiss fluorescence microscope.

### Flow cytometry analysis and cell sorting

Dissociated cells from E11.5 AGM, E11.5 FL, and E9.5 YS and cultured AGM explants were suspended in cold PEB buffer containing 2% FBS and passed through 40-μm cell strainers to obtain single-cell suspensions that were subjected to antibody staining. The following antibodies were used: AGMs cell sorting: PE-conjugated anti-CD31 (eBioscience, 12-0311-81) and APC-conjugated anti–c-Kit (eBioscience, 17-1171-81). AGM cell quantification: PE-conjugated anti-CD31 (eBioscience, 12-0311-81) and APC-conjugated anti–c-Kit (eBioscience,17-1171-81). YS cell quantification: PE-conjugated antibody CD31 (eBioscience, 12-0311-81), APC-conjugated anti–c-Kit (eBioscience, 17-1171-81), biotin-conjugated mouse lineage panel (BD Pharmingen, 559971), and APC-Cy7–conjugated streptavidin (BD Pharmingen). FL cell quantification: PE-conjugated anti-CD45 (BioLegend, 103106) and APC-conjugated anti–Ter-119 (BioLegend, 116212). AGM rescue experiments: PE-conjugated anti-CD31 (eBioscience, 12-0311-81), FITC-conjugated anti-CD31 (BioLegend, 102405), PE/Cy7 anti-mouse CD31 (BioLegend, 102418), APC-conjugated anti–c-Kit (eBioscience, 17-1171-81), and PE anti-mouse c-kit (eBioscience). NSG transplantation: PE-Cy7-CD45.1 (eBioscience, 25045382), FITC-CD45.2 (BioLegend, 109806), APC–Gr-1 (eBioscience, 17593182), PE-CD11b (eBioscience, 12011285), APC-Cy7-B220 (eBioscience, 47045282), PerCP-Cy5.5-CD3e (eBioscience, 45003182), Biotin Mouse Lineage Panel (BD Pharmingen, 559971), and APC-Cy7–conjugated streptavidin (BD Pharmingen). EB analysis: PE-conjugated anti-CD45 (BioLegend, 103106) and FITC-CD41 (Invitrogen, MA5-28367). All stainings were performed in PEB buffer (2 mM EDTA, 0.5% BSA, and 0.05% NaN_3_) using 1:100 dilution of each antibody for 20 min on ice. Where necessary, compensation was performed using the AbC Total Antibody Compensation Bead Kit (Thermo Fisher Scientific, A10497). DAPI (Invitrogen) staining was used to exclude dead cells. All cells were filtered through 40-μm filter before flow analysis. Flow cytometry data acquisition was performed on BD LSR II (BD) at Einstein Flow Cytometry Core Facility. Cell sorting was performed using a MoFlo XDP cell sorter (Beckman Coulter) at Einstein Human Stem Cell FACS and Xenotransplantation Facility. All data were analyzed using FlowJo v9.0.2 (Tree Star). Antibodies used are listed in table S2.

### Whole-embryo confocal microscopy

Embryos or YSs were fixed, stained, and analyzed following a published whole-mount fixation and multimarker staining protocol (*54*) using primary biotinylated rat anti-mouse CD31 (clone MEC13.3; 1:500; BD Pharmingen, 553371) and purified rat anti-mouse CD117 (clone 2b8; 1:500; BD Pharmingen, 553352) and secondary Alexa Fluor 647 goat anti-rat immunoglobulin G (lgG) (H+L) (1:5000; Invitrogen, A21247) and streptavidin Alexa Fluor 488 conjugated (1:5000; Invitrogen, S32354). Embryos were mounted in a 1:2 mix of benzyl alcohol (Sigma-Aldrich, 402834) and benzyl benzoate (Sigma-Aldrich, B6630) (BABB) in specialized Fast Well (Grace Bio-Lab, 664113) to increase the transparency of tissues and imaged with a Leica confocal microscope (SP8) at Einstein Analytical Imaging Facility. 3D reconstructions were generated from Z stacks (50 to 200 optical sections) using Fiji software. YSs were mounted in BABB and extended on the microscope slides (Thermo Fisher Scientific, 1255015), covered with cover slides, and imaged with Zeiss fluorescence microscope.

### EB formation and flow analysis

Tet1/2/3 TKO and wild-type V6.5 mouse ESCs were differentiated to EBs as described before (*17*). Day 12 EBs were dissociated into single cells by trypsinization, suspended into PEB buffer, labeled with PE-CD45 (BioLegend, 103106) and FITC-CD41 (Invitrogen, MA5-28367), and subjected to flow cytometry analysis.

### Apoptosis, proliferation, and cell cycle analysis of cells in AGM

Analysis of apoptotic cells in AGM was performed using the FITC annexin V Apoptosis Detection Kit I (BD Pharmingen, 556547) following the manufacturer’s protocol. Briefly, AGMs from E11.5 embryos were isolated and cultured for 4 days followed by dissociation into single cells and staining with PE-conjugated anti-mouse CD31 (eBioscience, 12-0311-81), APC-conjugated anti-CD117 (c-Kit) (eBioscience, 17-1171-81), and FITC annexin V and PI. Cells were subjected to flow cytometry analysis using BD LSR II. Analysis of cell proliferation in AGM was performed using the Click-iT EdU Alexa Fluor 647 Flow Cytometry Assay Kit (Invitrogen, C10424) following the manufacturer’s protocols. Briefly, three AGMs isolated from E11.5 embryos of the same genotype were mixed and dissociated into single cells and incubated with 10 μM EdU for 1.5 hours and then stained with FITC-conjugated anti-mouse CD31 (BioLegend, 102405). After fixation and permeabilization, cells were processed for Click-iT reaction. Stained cells were labelled with PE-conjugated anti-mouse CD117 and subjected to flow cytometry analysis using BD LSR II.

### RNA isolation and real-time PCR analysis

RNA was extracted from embryos or sorted cells using the Total RNA extraction Kit I (QIAGEN, R6834-02) and subjected to cDNA synthesis using SuperScript III First (Invitrogen, 18080-400). Real-time quantitative PCR was performed using the Fast SYBR Green Master Mix (Applied Biosystems, 4335612) in a QuantStudio 6 Flex Real-Time PCR system or a StepOnePlus Real-Time PCR system following the standard protocols using primers in table S1. Relative gene expression was calculated by comparative Ct method and normalized to *Gapdh* expression.

### Dot blot for 5hmC

DNA isolated from E11.5 *Tet1/2/3^f/f^;R26^+/CreER^* and littermate control embryos (tamoxifen at E7.5) were subjected to dot blot using anti-5hmC antibody (Active Motif, 39770) and methylene blue staining [0.02% (w/v) in 0.3 M sodium acetate (pH 5.5)] as described previously (*10*, *43*). 5hmC and methylene blue signal intensities were quantified by ImageJ software. 5hmC signal intensity was normalized to methylene blue signal intensity and plotted.

### Gene expression profiling by RNA-seq and data analysis

HSPCs and ECs were sorted from ~30 pooled E11.5 AGMs of same genotype using PE-CD31, APC–c-Kit, and DAPI. RNA was extracted using the QIAGEN AllPrep DNA/RNA Micro Kit (QIAGEN, 80284). RNA-seq libraries (three technical replicates) were prepared by first amplifying RNA using the SMART-Seq v4 Ultra Low Input RNA Kit for Sequencing (Takara, 634889) and then making libraries using the Nextera XT DNA Library Preparation Kit (illumine, FC-131-1024, 15055293) and Nextera XT Index Kit (FC-131-1001) following the manufacturer’s protocols. Libraries were analyzed by Qubit and bioanalyzer and then subjected to 75-bp paired-end sequencing (Illumina NextSeq 500 platform) at the Einstein Epigenomics Core. This generated 36 to 74 million read pairs per sample. The reads were trimmed using the TrimGalore (v0.4.1; https://github.com/FelixKrueger/TrimGalore) to remove adaptors and then mapped to the mouse genome (mm10) by TopHat2 software (v2.0.13) with default parameters. The read pair numbers mapped to individual genes in the Refseq gene annotation [downloaded from the University of California Santa Cruz (UCSC) genome browser] were calculated with the software HTseq (v0.6.1) using “--stranded = no” parameter. The fragments per kilobase of transcript per million (FPKMs) were calculated using the Cufflinks package (v2.2.1). Read counts of the genes with FPKM > 1 were imported to the DESeq2 software for differential expression. False discovery rate (FDR) < 0.05 and |fold change| > 1.5 were used for selecting DEGs between wild-type and knockout samples. GO analysis was performed by DAVID (https://david.ncifcrf.gov/tools.jsp) and ToppGene (https://toppgene.cchmc.org/). The expression patterns of DEGs were identified by *k*-means clustering and displayed by heatmap. Published mouse bone marrow Tet2 ChIP-seq data were downloaded from Gene Expression Omnibus database (accession number GSE115972) (*48*), and Tet2 peaks were called using MACS2 by default options, with corresponding IgG as control. Peaks were annotated to genes by R package “ChIPseeker.” Promoters were defined as ±2 kb of the TSS. Transcription factors (TFs) were screened out from Tet2 promoter-bound DEGs, taking all mouse TFs from the animal transcription factor database (AnimalTFDB 3.0) (*55*) as background. To construct transcriptional regulatory network, the regulation of TFs to DEGs was identified taking TF gene pairs from TRRUST database (v2) (*56*) as reference. Last, the network data were organized by Python script and visualized by Cytoscape (v3.3.0) (*57*).

### WGBS library preparation, sequencing, and data analysis

Library preparation and sequencing: For WGBS library construction, HSPCs and ECs were sorted from ~30 pooled E11.5 AGMs of same genotype using PE-CD31, APC–c-Kit, and DAPI. DNA was extracted using the RNeasy Plus Miro Kit (QIAGEN, 74034). DNA (10 ng) was bisulfite-treated, and WGBS libraries were constructed using the Pico Methyl-Seq Library Prep Kit for IIIumina-based sequencing (Zymo Research, D5455) following the manufacturer’s protocols. Libraries were subjected to 150-bp paired-end sequencing using Novagene’s IIIumina HiSeq 4000 platform. Data quality control and read mapping: First, Trim Galore (v0.3.7) was used to trim the first four unsteady bases from 5′ regions of both strands. Then, low-quality ends of reads were trimmed by default parameter “-q 20.” For adapter trimming, per the Pico Methyl-Seq Library Prep Kit instructions, the sequence “GATCGGAAGAGC” was used instead of the Illumina default adapters (AGATCGGAAGAGC). In addition, according to the kit protocol, the methylation conversion rates decrease after 50 bp, so all sequences that are longer than 50 bp were cropped to 50 bp by Trimmomatic (v0.36). The filtered reads were mapped to mm10 mouse genome by Bismark (v0.18.1) (*58*) with the option “--non_directional.” Calling DMRs: MethPipe (v3.4.3) (*59*) was used to convert the output from Bismark mapper and compute methylation levels at each cytosine site along the genome. DMRfind from Methylpy (v1.4.0) (*60*) was used to identify differentially methylated sites (DMSs) or DMRs. For DMSs, threshold was set as *P* < 0.01 and methylation difference ≥ 20%; and for DMRs, at least three DMSs were required to be in a region and the maximal distance between two adjacent sites should be less than 300 bp, FDR < 0.01, and methylation difference ≥ 20%. Methylpy was used to convert the methylation levels to bigwig files for visualization by Integrative Genomics Viewer (IGV). R package methimpute (v1.8.0) was used to bin the genome into 1000-bp tiles and to calculate the methylated counts and total counts for each tile. Genome-wide methylation distribution was shown by density plot and cumulative plot by R using 1000-bp tiles (at least three CpG sites at each tile). DMRs were annotated to genomic regions by R package “ChIPseeker” (v1.16.1). Composite plot of methylation differences around DMRs was drawn by deepTools (v3.1.0). The average methylation profile was calculated for 5-kb upstream and downstream of the center of each DMR. To identify enhancer DMRs, public PreHSC and endothelium H3K4me3 and H3K4me1 ChIP-seq datasets (accession number GSE135601) (*51*) were used to distinguish promoter (H3K4me3 high) and enhancer (H3K4me1 high and H3K4me3 low) DMRs, respectively. Methylation canyons were identified by “hmr” from MethPipe software. CpG methylation levels and Tet1 binding signals at canyons were visualized in the same plot.

### Quantification and statistical analyses

Student’s *t* test was used to calculate significant differences between two groups. Data are presented as means ± SEM. *P* < 0.05 was considered statically significant. GraphPad Prism 8 software was used for data analysis. Statistical methods for analysis of genome-wide datasets involving RNA-seq and WGBS are explained in detail under the respective sections as part of the detailed methods.

## Notes

View/request a protocol for this paper from *Bio-protocol*.

## References

[1] Z. D. Smith, A. Meissner, DNA methylation: Roles in mammalian development. Nat. Rev. Genet. 14, 204–220 (2013). 23400093 10.1038/nrg3354

[2] W. A. Pastor, L. Aravind, A. Rao, TETonic shift: Biological roles of TET proteins in DNA demethylation and transcription. Nat. Rev. Mol. Cell Biol. 14, 341–356 (2013). 23698584 10.1038/nrm3589 PMC3804139

[3] M. Tahiliani, K. P. Koh, Y. Shen, W. A. Pastor, H. Bandukwala, Y. Brudno, S. Agarwal, L. M. Iyer, D. R. Liu, L. Aravind, A. Rao, Conversion of 5-methylcytosine to 5-hydroxymethylcytosine in mammalian DNA by MLL partner TET1. Science 324, 930–935 (2009). 19372391 10.1126/science.1170116 PMC2715015

[4] Y. F. He, B. Z. Li, Z. Li, P. Liu, Y. Wang, Q. Tang, J. Ding, Y. Jia, Z. Chen, L. Li, Y. Sun, X. Li, Q. Dai, C. X. Song, K. Zhang, C. He, G. L. Xu, Tet-mediated formation of 5-carboxylcytosine and its excision by TDG in mammalian DNA. Science 333, 1303–1307 (2011). 21817016 10.1126/science.1210944 PMC3462231

[5] S. Ito, L. Shen, Q. Dai, S. C. Wu, L. B. Collins, J. A. Swenberg, C. He, Y. Zhang, Tet proteins can convert 5-methylcytosine to 5-formylcytosine and 5-carboxylcytosine. Science 333, 1300–1303 (2011). 21778364 10.1126/science.1210597 PMC3495246

[6] H. Wu, Y. Zhang, Reversing DNA methylation: Mechanisms, genomics, and biological functions. Cell 156, 45–68 (2014). 24439369 10.1016/j.cell.2013.12.019 PMC3938284

[7] T. P. Gu, F. Guo, H. Yang, H. P. Wu, G. F. Xu, W. Liu, Z. G. Xie, L. Shi, X. He, S. G. Jin, K. Iqbal, Y. G. Shi, Z. Deng, P. E. Szabo, G. P. Pfeifer, J. Li, G. L. Xu, The role of Tet3 DNA dioxygenase in epigenetic reprogramming by oocytes. Nature 477, 606–610 (2011). 21892189 10.1038/nature10443

[8] K. P. Koh, A. Yabuuchi, S. Rao, Y. Huang, K. Cunniff, J. Nardone, A. Laiho, M. Tahiliani, C. A. Sommer, G. Mostoslavsky, R. Lahesmaa, S. H. Orkin, S. J. Rodig, G. Q. Daley, A. Rao, Tet1 and Tet2 regulate 5-hydroxymethylcytosine production and cell lineage specification in mouse embryonic stem cells. Cell Stem Cell 8, 200–213 (2011). 21295276 10.1016/j.stem.2011.01.008 PMC3134318

[9] S. Ito, A. C. D’Alessio, O. V. Taranova, K. Hong, L. C. Sowers, Y. Zhang, Role of Tet proteins in 5mC to 5hmC conversion, ES-cell self-renewal and inner cell mass specification. Nature 466, 1129–1133 (2010). 20639862 10.1038/nature09303 PMC3491567

[10] M. M. Dawlaty, K. Ganz, B. E. Powell, Y. C. Hu, S. Markoulaki, A. W. Cheng, Q. Gao, J. Kim, S. W. Choi, D. C. Page, R. Jaenisch, Tet1 is dispensable for maintaining pluripotency and its loss is compatible with embryonic and postnatal development. Cell Stem Cell 9, 166–175 (2011). 21816367 10.1016/j.stem.2011.07.010 PMC3154739

[11] M. M. Dawlaty, A. Breiling, T. Le, G. Raddatz, M. I. Barrasa, A. W. Cheng, Q. Gao, B. E. Powell, Z. Li, M. Xu, K. F. Faull, F. Lyko, R. Jaenisch, Combined deficiency of Tet1 and Tet2 causes epigenetic abnormalities but is compatible with postnatal development. Dev. Cell 24, 310–323 (2013). 23352810 10.1016/j.devcel.2012.12.015 PMC3574201

[12] R. Khoueiry, A. Sohni, B. Thienpont, X. Luo, J. V. Velde, M. Bartoccetti, B. Boeckx, A. Zwijsen, A. Rao, D. Lambrechts, K. P. Koh, Lineage-specific functions of TET1 in the postimplantation mouse embryo. Nat. Genet. 49, 1061–1072 (2017). 28504700 10.1038/ng.3868 PMC6033328

[13] S. Yamaguchi, K. Hong, R. Liu, L. Shen, A. Inoue, D. Diep, K. Zhang, Y. Zhang, Tet1 controls meiosis by regulating meiotic gene expression. Nature 492, 443–447 (2012). 23151479 10.1038/nature11709 PMC3528851

[14] Z. Li, X. Cai, C. L. Cai, J. Wang, W. Zhang, B. E. Petersen, F. C. Yang, M. Xu, Deletion of Tet2 in mice leads to dysregulated hematopoietic stem cells and subsequent development of myeloid malignancies. Blood 118, 4509–4518 (2011). 21803851 10.1182/blood-2010-12-325241 PMC3952630

[15] M. Ko, H. S. Bandukwala, J. An, E. D. Lamperti, E. C. Thompson, R. Hastie, A. Tsangaratou, K. Rajewsky, S. B. Koralov, A. Rao, Ten-Eleven-Translocation 2 (TET2) negatively regulates homeostasis and differentiation of hematopoietic stem cells in mice. Proc. Natl. Acad. Sci. U.S.A. 108, 14566–14571 (2011). 21873190 10.1073/pnas.1112317108 PMC3167529

[16] X. Li, X. Yue, W. A. Pastor, L. Lin, R. Georges, L. Chavez, S. M. Evans, A. Rao, Tet proteins influence the balance between neuroectodermal and mesodermal fate choice by inhibiting Wnt signaling. Proc. Natl. Acad. Sci. U.S.A. 113, E8267–E8276 (2016). 27930333 10.1073/pnas.1617802113 PMC5187696

[17] M. M. Dawlaty, A. Breiling, T. Le, M. I. Barrasa, G. Raddatz, Q. Gao, B. E. Powell, A. W. Cheng, K. F. Faull, F. Lyko, R. Jaenisch, Loss of Tet enzymes compromises proper differentiation of embryonic stem cells. Dev. Cell 29, 102–111 (2014). 24735881 10.1016/j.devcel.2014.03.003 PMC4035811

[18] H. Q. Dai, B. A. Wang, L. Yang, J. J. Chen, G. C. Zhu, M. L. Sun, H. Ge, R. Wang, D. L. Chapman, F. Tang, X. Sun, G. L. Xu, TET-mediated DNA demethylation controls gastrulation by regulating Lefty-Nodal signalling. Nature 538, 528–532 (2016). 27760115 10.1038/nature20095

[19] S. Fang, J. Li, Y. Xiao, M. Lee, L. Guo, W. Han, T. Li, M. C. Hill, T. Hong, W. Mo, R. Xu, P. Zhang, F. Wang, J. Chang, Y. Zhou, D. Sun, J. F. Martin, Y. Huang, Tet inactivation disrupts YY1 binding and long-range chromatin interactions during embryonic heart development. Nat. Commun. 10, 4297 (2019). 31541101 10.1038/s41467-019-12325-z PMC6754421

[20] L. Cimmino, O. Abdel-Wahab, R. L. Levine, I. Aifantis, TET family proteins and their role in stem cell differentiation and transformation. Cell Stem Cell 9, 193–204 (2011). 21885017 10.1016/j.stem.2011.08.007 PMC3244690

[21] E. Dzierzak, N. A. Speck, Of lineage and legacy: The development of mammalian hematopoietic stem cells. Nat. Immunol. 9, 129–136 (2008). 18204427 10.1038/ni1560 PMC2696344

[22] T. Yamane, Mouse Yolk Sac Hematopoiesis. Front. Cell Dev. Biol. 6, 80 (2018). 30079337 10.3389/fcell.2018.00080 PMC6062755

[23] G. Costa, V. Kouskoff, G. Lacaud, Origin of blood cells and HSC production in the embryo. Trends Immunol. 33, 215–223 (2012). 22365572 10.1016/j.it.2012.01.012

[24] A. Medvinsky, E. Dzierzak, Definitive hematopoiesis is autonomously initiated by the AGM region. Cell 86, 897–906 (1996). 8808625 10.1016/s0092-8674(00)80165-8

[25] A. L. Medvinsky, N. L. Samoylina, A. M. Muller, E. A. Dzierzak, An early pre-liver intraembryonic source of CFU-S in the developing mouse. Nature 364, 64–67 (1993). 8316298 10.1038/364064a0

[26] A. C. Zovein, J. J. Hofmann, M. Lynch, W. J. French, K. A. Turlo, Y. Yang, M. S. Becker, L. Zanetta, E. Dejana, J. C. Gasson, M. D. Tallquist, M. L. Iruela-Arispe, Fate tracing reveals the endothelial origin of hematopoietic stem cells. Cell Stem Cell 3, 625–636 (2008). 19041779 10.1016/j.stem.2008.09.018 PMC2631552

[27] J. Y. Bertrand, N. C. Chi, B. Santoso, S. Teng, D. Y. Stainier, D. Traver, Haematopoietic stem cells derive directly from aortic endothelium during development. Nature 464, 108–111 (2010). 20154733 10.1038/nature08738 PMC2858358

[28] J. C. Boisset, W. van Cappellen, C. Andrieu-Soler, N. Galjart, E. Dzierzak, C. Robin, In vivo imaging of haematopoietic cells emerging from the mouse aortic endothelium. Nature 464, 116–120 (2010). 20154729 10.1038/nature08764

[29] G. Swiers, C. Rode, E. Azzoni, M. F. de Bruijn, A short history of hemogenic endothelium. Blood Cells Mol. Dis. 51, 206–212 (2013). 24095001 10.1016/j.bcmd.2013.09.005 PMC4700588

[30] M. J. Chen, T. Yokomizo, B. M. Zeigler, E. Dzierzak, N. A. Speck, Runx1 is required for the endothelial to haematopoietic cell transition but not thereafter. Nature 457, 887–891 (2009). 19129762 10.1038/nature07619 PMC2744041

[31] E. de Pater, P. Kaimakis, C. S. Vink, T. Yokomizo, T. Yamada-Inagawa, R. van der Linden, P. S. Kartalaei, S. A. Camper, N. Speck, E. Dzierzak, Gata2 is required for HSC generation and survival. J. Exp. Med. 210, 2843–2850 (2013). 24297996 10.1084/jem.20130751 PMC3865477

[32] X. Gao, K. D. Johnson, Y. I. Chang, M. E. Boyer, C. N. Dewey, J. Zhang, E. H. Bresnick, Gata2 cis-element is required for hematopoietic stem cell generation in the mammalian embryo. J. Exp. Med. 210, 2833–2842 (2013). 24297994 10.1084/jem.20130733 PMC3865483

[33] H. Beauchemin, T. Moroy, Multifaceted actions of GFI1 and GFI1B in hematopoietic stem cell self-renewal and lineage commitment. Front. Genet. 11, 591099 (2020). 33193732 10.3389/fgene.2020.591099 PMC7649360

[34] H. Celik, A. Kramer, G. A. Challen, DNA methylation in normal and malignant hematopoiesis. Int. J. Hematol. 103, 617–626 (2016). 26943352 10.1007/s12185-016-1957-7

[35] J. J. Trowbridge, J. W. Snow, J. Kim, S. H. Orkin, DNA methyltransferase 1 is essential for and uniquely regulates hematopoietic stem and progenitor cells. Cell Stem Cell 5, 442–449 (2009). 19796624 10.1016/j.stem.2009.08.016 PMC2767228

[36] A. M. Broske, L. Vockentanz, S. Kharazi, M. R. Huska, E. Mancini, M. Scheller, C. Kuhl, A. Enns, M. Prinz, R. Jaenisch, C. Nerlov, A. Leutz, M. A. Andrade-Navarro, S. E. Jacobsen, F. Rosenbauer, DNA methylation protects hematopoietic stem cell multipotency from myeloerythroid restriction. Nat. Genet. 41, 1207–1215 (2009). 19801979 10.1038/ng.463

[37] G. A. Challen, D. Sun, M. Jeong, M. Luo, J. Jelinek, J. S. Berg, C. Bock, A. Vasanthakumar, H. Gu, Y. Xi, S. Liang, Y. Lu, G. J. Darlington, A. Meissner, J. P. Issa, L. A. Godley, W. Li, M. A. Goodell, Dnmt3a is essential for hematopoietic stem cell differentiation. Nat. Genet. 44, 23–31 (2011). 22138693 10.1038/ng.1009 PMC3637952

[38] G. A. Challen, D. Sun, A. Mayle, M. Jeong, M. Luo, B. Rodriguez, C. Mallaney, H. Celik, L. Yang, Z. Xia, S. Cullen, J. Berg, Y. Zheng, G. J. Darlington, W. Li, M. A. Goodell, Dnmt3a and Dnmt3b have overlapping and distinct functions in hematopoietic stem cells. Cell Stem Cell 15, 350–364 (2014). 25130491 10.1016/j.stem.2014.06.018 PMC4163922

[39] L. Cimmino, M. M. Dawlaty, D. Ndiaye-Lobry, Y. S. Yap, S. Bakogianni, Y. Yu, S. Bhattacharyya, R. Shaknovich, H. Geng, C. Lobry, J. Mullenders, B. King, T. Trimarchi, B. Aranda-Orgilles, C. Liu, S. Shen, A. K. Verma, R. Jaenisch, I. Aifantis, TET1 is a tumor suppressor of hematopoietic malignancy. Nat. Immunol. 16, 653–662 (2015). 25867473 10.1038/ni.3148 PMC4545281

[40] O. Abdel-Wahab, A. Mullally, C. Hedvat, G. Garcia-Manero, J. Patel, M. Wadleigh, S. Malinge, J. Yao, O. Kilpivaara, R. Bhat, K. Huberman, S. Thomas, I. Dolgalev, A. Heguy, E. Paietta, M. M. Le Beau, M. Beran, M. S. Tallman, B. L. Ebert, H. M. Kantarjian, R. M. Stone, D. G. Gilliland, J. D. Crispino, R. L. Levine, Genetic characterization of TET1, TET2, and TET3 alterations in myeloid malignancies. Blood 114, 144–147 (2009). 19420352 10.1182/blood-2009-03-210039 PMC2710942

[41] K. Moran-Crusio, L. Reavie, A. Shih, O. Abdel-Wahab, D. Ndiaye-Lobry, C. Lobry, M. E. Figueroa, A. Vasanthakumar, J. Patel, X. Zhao, F. Perna, S. Pandey, J. Madzo, C. Song, Q. Dai, C. He, S. Ibrahim, M. Beran, J. Zavadil, S. D. Nimer, A. Melnick, L. A. Godley, I. Aifantis, R. L. Levine, Tet2 loss leads to increased hematopoietic stem cell self-renewal and myeloid transformation. Cancer Cell 20, 11–24 (2011). 21723200 10.1016/j.ccr.2011.06.001 PMC3194039

[42] F. Pan, T. S. Wingo, Z. Zhao, R. Gao, H. Makishima, G. Qu, L. Lin, M. Yu, J. R. Ortega, J. Wang, A. Nazha, L. Chen, B. Yao, C. Liu, S. Chen, O. Weeks, H. Ni, B. L. Phillips, S. Huang, J. Wang, C. He, G. M. Li, T. Radivoyevitch, I. Aifantis, J. P. Maciejewski, F. C. Yang, P. Jin, M. Xu, Tet2 loss leads to hypermutagenicity in haematopoietic stem/progenitor cells. Nat. Commun. 8, 15102 (2017). 28440315 10.1038/ncomms15102 PMC5414116

[43] K. Ito, J. Lee, S. Chrysanthou, Y. Zhao, K. Josephs, H. Sato, J. Teruya-Feldstein, D. Zheng, M. M. Dawlaty, K. Ito, Non-catalytic roles of Tet2 are essential to regulate hematopoietic stem and progenitor cell homeostasis. Cell Rep. 28, 2480–2490.e4 (2019). 31484061 10.1016/j.celrep.2019.07.094 PMC6750732

[44] J. An, E. Gonzalez-Avalos, A. Chawla, M. Jeong, I. F. Lopez-Moyado, W. Li, M. A. Goodell, L. Chavez, M. Ko, A. Rao, Acute loss of TET function results in aggressive myeloid cancer in mice. Nat. Commun. 6, 10071 (2015). 26607761 10.1038/ncomms10071 PMC4674670

[45] X. Liu, X. Jia, H. Yuan, K. Ma, Y. Chen, Y. Jin, M. Deng, W. Pan, S. Chen, Z. Chen, H. de The, L. I. Zon, Y. Zhou, J. Zhou, J. Zhu, DNA methyltransferase 1 functions through C/ebpa to maintain hematopoietic stem and progenitor cells in zebrafish. J. Hematol. Oncol. 8, 15 (2015). 25886310 10.1186/s13045-015-0115-7 PMC4372312

[46] A. V. Gore, B. Athans, J. R. Iben, K. Johnson, V. Russanova, D. Castranova, V. N. Pham, M. G. Butler, L. Williams-Simons, J. T. Nichols, E. Bresciani, B. Feldman, C. B. Kimmel, P. P. Liu, B. M. Weinstein, Epigenetic regulation of hematopoiesis by DNA methylation. eLife 5, e11813 (2016). 26814702 10.7554/eLife.11813 PMC4744183

[47] C. Li, Y. Lan, L. Schwartz-Orbach, E. Korol, M. Tahiliani, T. Evans, M. G. Goll, Overlapping requirements for Tet2 and Tet3 in normal development and hematopoietic stem cell emergence. Cell Rep. 12, 1133–1143 (2015). 26257178 10.1016/j.celrep.2015.07.025 PMC4545447

[48] K. D. Rasmussen, I. Berest, S. Keβler, K. Nishimura, L. Simón-Carrasco, G. S. Vassiliou, M. T. Pedersen, J. Christensen, J. B. Zaugg, K. Helin, TET2 binding to enhancers facilitates transcription factor recruitment in hematopoietic cells. Genome Res. 29, 564–575 (2019). 30796038 10.1101/gr.239277.118 PMC6442383

[49] K. Williams, J. Christensen, M. T. Pedersen, J. V. Johansen, P. A. Cloos, J. Rappsilber, K. Helin, TET1 and hydroxymethylcytosine in transcription and DNA methylation fidelity. Nature 473, 343–348 (2011). 21490601 10.1038/nature10066 PMC3408592

[50] Z. Zhao, L. Chen, M. M. Dawlaty, F. Pan, O. Weeks, Y. Zhou, Z. Cao, H. Shi, J. Wang, L. Lin, S. Chen, W. Yuan, Z. Qin, H. Ni, S. D. Nimer, F. C. Yang, R. Jaenisch, P. Jin, M. Xu, Combined Loss of Tet1 and Tet2 Promotes B Cell, but Not Myeloid Malignancies, in Mice. Cell Rep. 13, 1692–1704 (2015). 26586431 10.1016/j.celrep.2015.10.037 PMC4764044

[51] P. Gao, C. Chen, E. D. Howell, Y. Li, J. Tober, Y. Uzun, B. He, L. Gao, Q. Zhu, A. F. Siekmann, N. A. Speck, K. Tan, Transcriptional regulatory network controlling the ontogeny of hematopoietic stem cells. Genes Dev. 34, 950–964 (2020). 32499402 10.1101/gad.338202.120 PMC7328518

[52] L. Ge, R. P. Zhang, F. Wan, D. Y. Guo, P. Wang, L. X. Xiang, J. Z. Shao, TET2 plays an essential role in erythropoiesis by regulating lineage-specific genes via DNA oxidative demethylation in a zebrafish model. Mol. Cell. Biol. 34, 989–1002 (2014). 24396069 10.1128/MCB.01061-13 PMC3958037

[53] L. T. Vo, M. A. Kinney, X. Liu, Y. Zhang, J. Barragan, P. M. Sousa, D. K. Jha, A. Han, M. Cesana, Z. Shao, T. E. North, S. H. Orkin, S. Doulatov, J. Xu, G. Q. Daley, Regulation of embryonic haematopoietic multipotency by EZH1. Nature 553, 506–510 (2018). 29342143 10.1038/nature25435 PMC5785461

[54] T. Yokomizo, T. Yamada-Inagawa, A. D. Yzaguirre, M. J. Chen, N. A. Speck, E. Dzierzak, Whole-mount three-dimensional imaging of internally localized immunostained cells within mouse embryos. Nat. Protoc. 7, 421–431 (2012). 22322215 10.1038/nprot.2011.441 PMC3629302

[55] H. Hu, Y. R. Miao, L. H. Jia, Q. Y. Yu, Q. Zhang, A. Y. Guo, AnimalTFDB 3.0: A comprehensive resource for annotation and prediction of animal transcription factors. Nucleic Acids Res. 47, D33–D38 (2019). 30204897 10.1093/nar/gky822 PMC6323978

[56] H. Han, J. W. Cho, S. Lee, A. Yun, H. Kim, D. Bae, S. Yang, C. Y. Kim, M. Lee, E. Kim, S. Lee, B. Kang, D. Jeong, Y. Kim, H. N. Jeon, H. Jung, S. Nam, M. Chung, J. H. Kim, I. Lee, TRRUST v2: An expanded reference database of human and mouse transcriptional regulatory interactions. Nucleic Acids Res. 46, D380–D386 (2018). 29087512 10.1093/nar/gkx1013 PMC5753191

[57] P. Shannon, A. Markiel, O. Ozier, N. S. Baliga, J. T. Wang, D. Ramage, N. Amin, B. Schwikowski, T. Ideker, Cytoscape: A software environment for integrated models of biomolecular interaction networks. Genome Res. 13, 2498–2504 (2003). 14597658 10.1101/gr.1239303 PMC403769

[58] F. Krueger, S. R. Andrews, Bismark: A flexible aligner and methylation caller for Bisulfite-Seq applications. Bioinformatics 27, 1571–1572 (2011). 21493656 10.1093/bioinformatics/btr167 PMC3102221

[59] Q. Song, B. Decato, E. E. Hong, M. Zhou, F. Fang, J. Qu, T. Garvin, M. Kessler, J. Zhou, A. D. Smith, A reference methylome database and analysis pipeline to facilitate integrative and comparative epigenomics. PLOS ONE 8, e81148 (2013). 24324667 10.1371/journal.pone.0081148 PMC3855694

[60] M. D. Schultz, Y. He, J. W. Whitaker, M. Hariharan, E. A. Mukamel, D. Leung, N. Rajagopal, J. R. Nery, M. A. Urich, H. Chen, S. Lin, Y. Lin, I. Jung, A. D. Schmitt, S. Selvaraj, B. Ren, T. J. Sejnowski, W. Wang, J. R. Ecker, Human body epigenome maps reveal noncanonical DNA methylation variation. Nature 523, 212–216 (2015). 26030523 10.1038/nature14465 PMC4499021

