# Supplementary material for: Tet-mediated DNA demethylation regulates specification of hematopoietic stem and progenitor cells during mammalian embryogenesis
Source: Sci Adv. 2022 Mar 2;8(9):eabm3470. doi: 10.1126/sciadv.abm3470 (PMC8890710; doi:10.1126/sciadv.abm3470)
Supplement: Supplementary file 1 — Figs. S1 to S7 Tables S1 and S2 [file sciadv.abm3470_sm.pdf]

Science Advances
AAAS

Supplementary Materials for

Tet-mediated DNA demethylation regulates specification of hematopoietic stem and progenitor cells during mammalian embryogenesis

Liyang Ma, Qin Tang, Xin Gao, Joun Lee, Run Lei, Masako Suzuki, Deyou Zheng, Keisuke Ito, Paul S. Frenette, Meelad M. Dawlaty*

*Corresponding author. Email: meelad.dawlaty@einsteinmed.org

Published 2 March 2022, *Sci. Adv.* **8**, eabm3470 (2022)
DOI: 10.1126/sciadv.abm3470

This PDF file includes:

Figs. S1 to S7
Tables S1 and S2

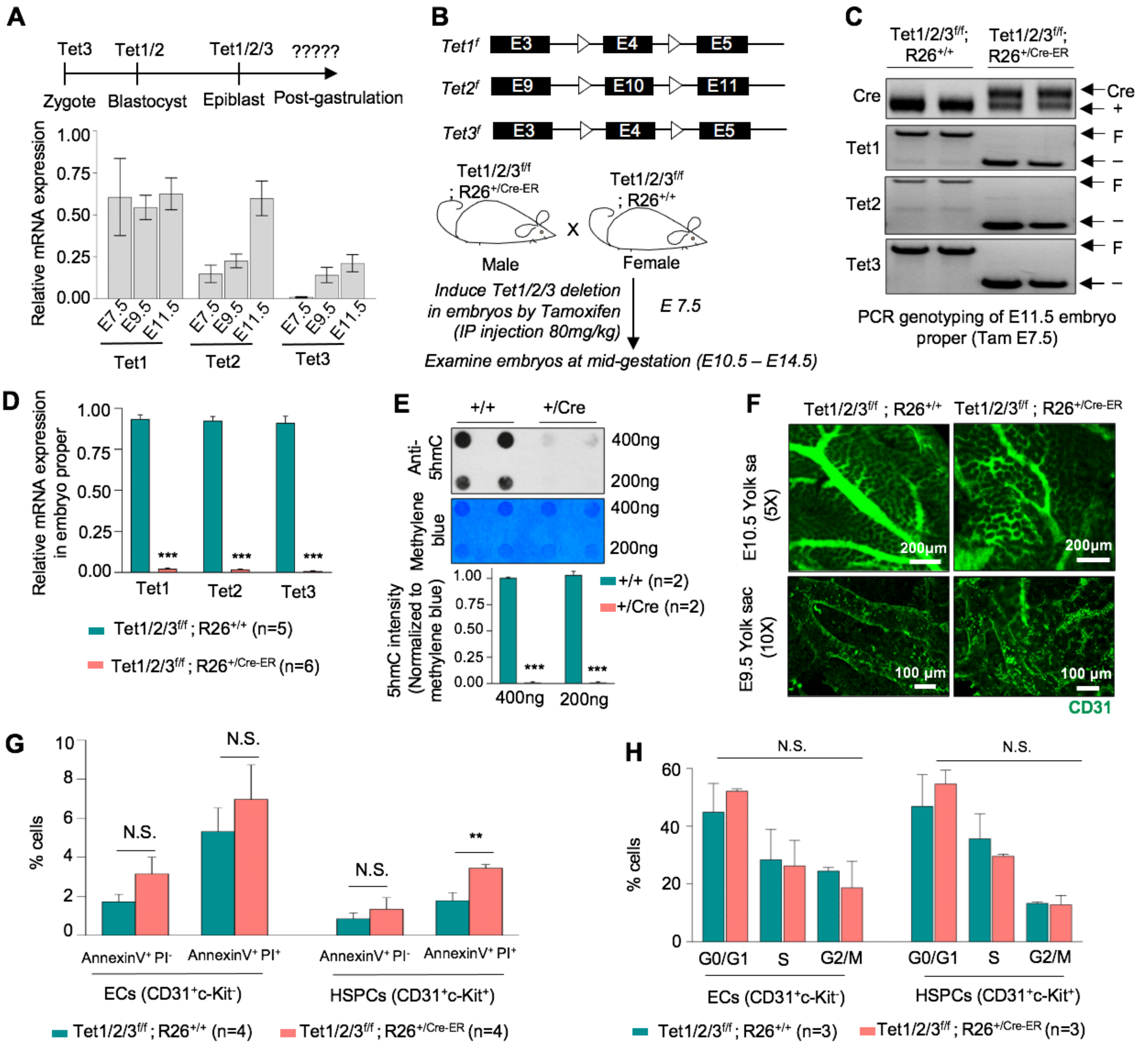

Fig. S1. Generation and characterization of *Tet1/2/3$^{f/f}$;R26$^{+/CreER}$* tamoxifen-inducible mice (A) Quantification of Tet1/2/3 mRNA expression in E7.5, E9.5 and E11.5 whole embryos by RT-qPCR. Data normalized to Gapdh. 8 embryos from 3 independent litters were analyzed for each time point. **(B)** Schematic of *Tet1/2/3* conditional alleles, and breeding and tamoxifen administration strategies for inducing *Tet1/2/3* deletion during embryogenesis. **(C)** PCR genotyping of *Rosa26-Cre*, *Tet1*, *Tet2* and *Tet3* alleles using DNA extracted from E11.5 embryo proper (tamoxifen treated at E7.5). **(D)** Quantification of Tet1/2/3 mRNA expression in E11.5 embryo proper by RT-qPCR. Data normalized to Gapdh. Embryos of each genotype from a total of 3 litters were analyzed. **(E)** 5hmC levels in DNA isolated from E11.5 embryo proper of indicated genotypes (tamoxifen treated E7.5) quantified by dot plot using anti 5hmC antibody. Methylene blue staining is used as loading control. 5hmC signal intensity is normalized to that of methylene blue and plotted. **(F)** Representative whole mount images of E9.5 and E10.5 Yolk sacs of indicated genotypes stained with CD31 to visualize vessels. **(G)** Quantification of apoptotic ECs and HSPCs by Annexin V and PI staining in E11.5 AGMs of indicated genotypes (treated with tamoxifen at E7.5). **(H)** Cell cycle analysis of CD31^{+}c-Kit^{+} HSPCs and CD31^{+}c-Kit^{-} ECs by EdU incorporation in E11.5 AGMs (treated with tamoxifen at E7.5). In all panels data presented as mean ± SEM. Statistically significant (*p<0.05, ** p<0.01, ***p<0.001). N.S. no significant change.

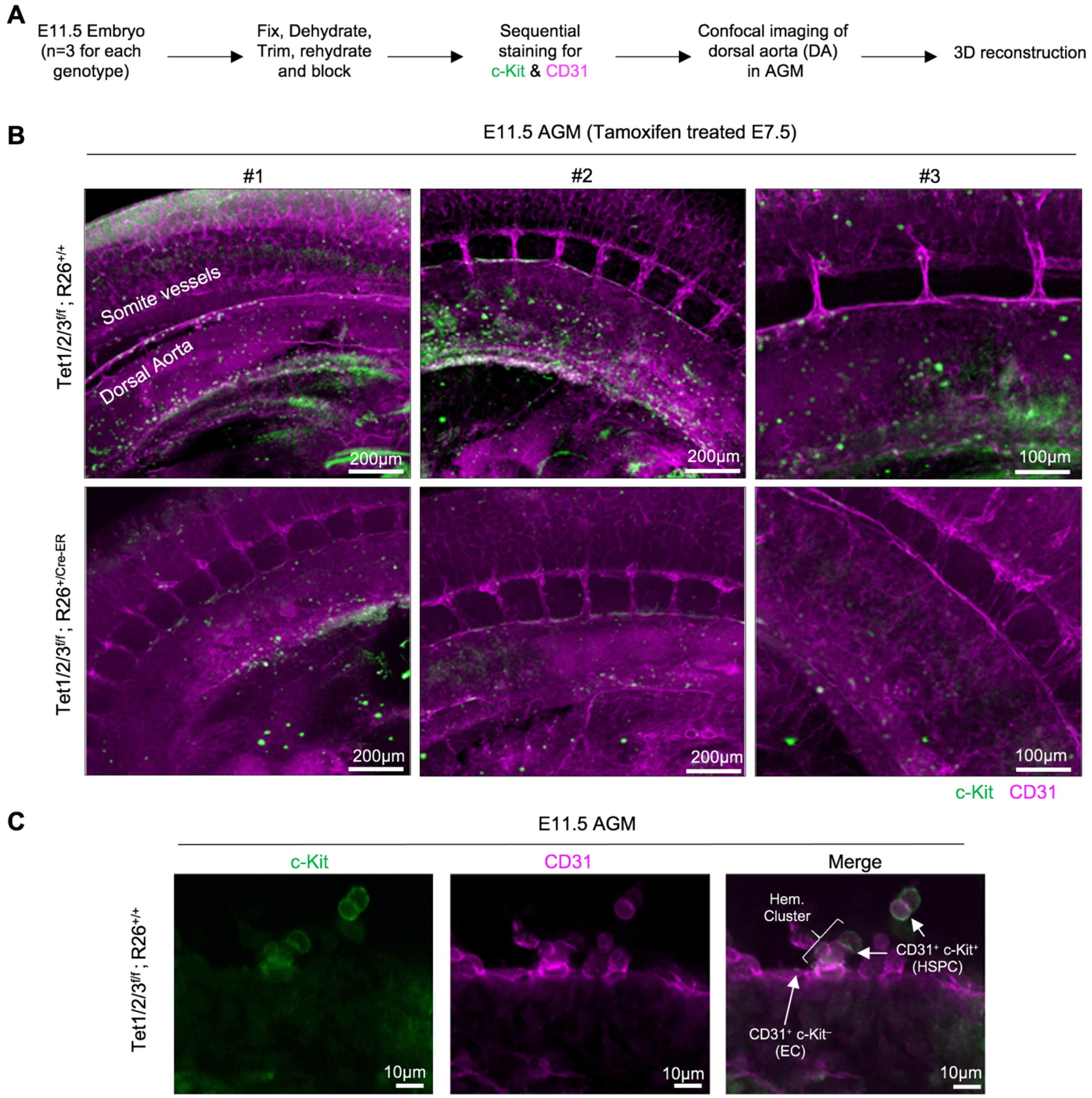

Fig. S2. Lack of CD31$^+$ c-Kit$^+$ hematopoietic clusters and floating HSPCs in AGMs of tamoxifen-treated *Tet1/2/3$^{f/f}$;R26$^{+/CreER}$* embryos. (A) Schematic of whole-mount 3D immunostaining of E11.5 AGMs. **(B)** Whole-mount 3D images of E11.5 AGMs of indicated genotypes stained with CD31 and c-Kit antibodies. CD31$^+$ ECs (magenta) and c-Kit$^+$ HSPC (green) within the dorsal aorta region are shown in AGM #1 & #2 at 10X and in AGM #3 at 20X magnification. **(C)** Images of CD31 and c-Kit double positive hematopoietic clusters shown at 100X magnification. HSPC, hematopoietic stem and progenitor cell; EC, endothelial cell; Hem. Cluster, hematopoietic cluster.

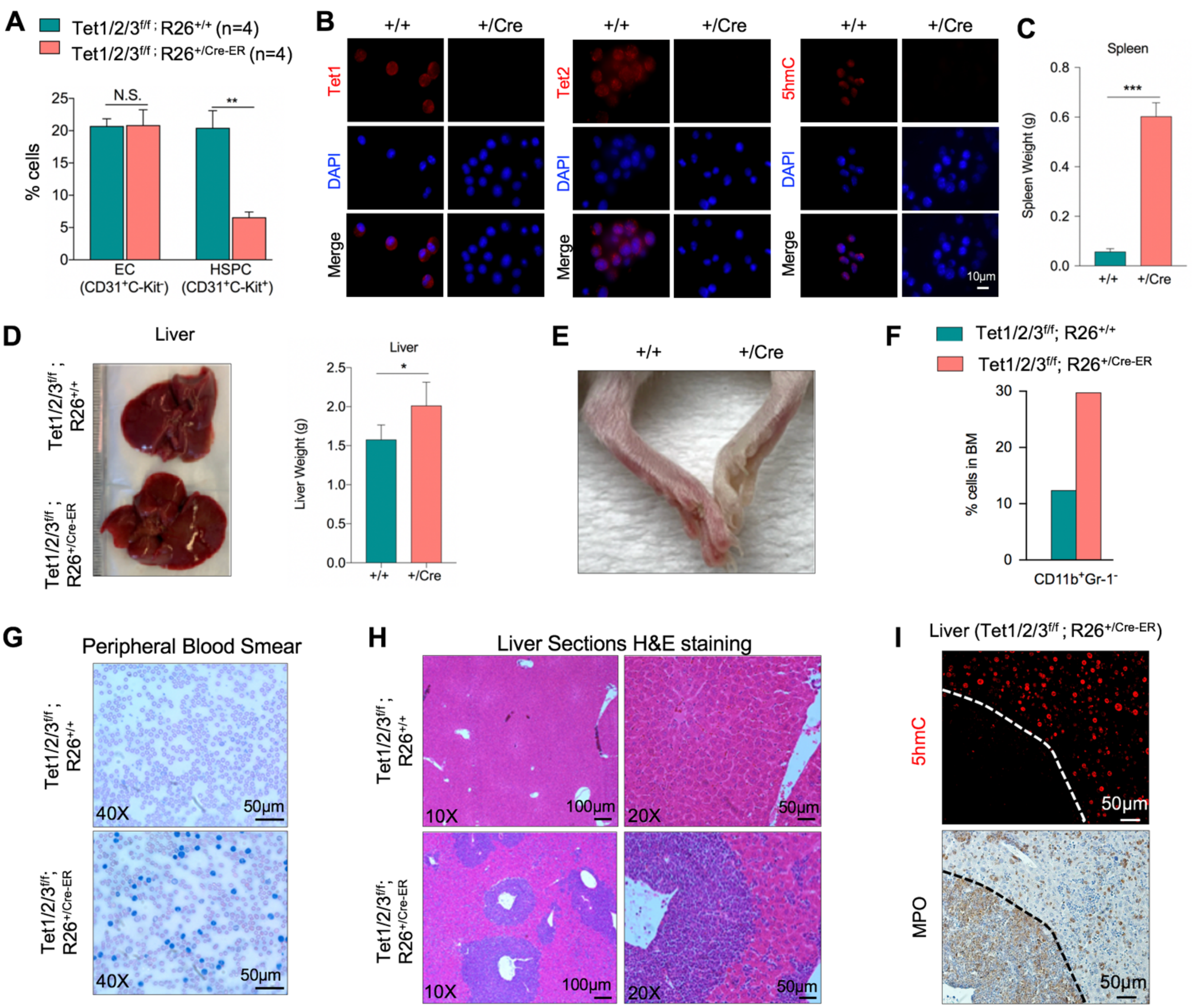

Fig. S3. Characterization of mice transplanted with Tet-deficient HSPCs. (A) Quantification of ECs and HSPCs in cultured E11.5 AGMs (isolated from E7.5-tamoxifen-treated embryos) of indicated genotypes by flow cytometry. Four independent AGMs of each genotype were cultured and analyzed. **(B)** Detection of Tet enzymes and 5hmC by immunofluorescence in cultured *Tet1/2/3$^{f/f}$;R26$^{+/+}$* and *Tet1/2/3$^{f/f}$;R26$^{+/CreER}$* HSPCs (isolated from E11.5 AGMs, tamoxifen treated E7.5) using anti Tet1, Tet2 and 5hmC antibodies. Nuclei are stained with Dapi. Note lack of Tet enzymes and 5hmC in Tet-deficient cells. **(C)** Quantification of spleen weights of 10-12-week post-transplantation NSG mice (n=4 of each genotype). **(D)** Gross images (left) and weights (rights) of livers of 10-12-week post-transplantation NSG mice (n=4 of each genotype). **(E)** Gross images of paws of 10-12-week post-transplantation NSG mice. Note the paleness indicative of anemia in paw of mouse transplanted with Tet-deficient AGMs. **(F)** Quantification of undifferentiated myeloid cells (CD11b^{+} Gr-1^{-}) in the bone morrow of 12-week post-transplantation NSG recipient mice (n=1 of each genotype). **(G)** May–Grünwald–Giemsa-stained peripheral blood smears of 11-week-post-transplantation recipient mice. **(H)** Hematoxylin and eosin staining of livers of 12-week-post-transplantation recipient mice. **(I)** Immunohistochemical staining of a liver section of 12-week post-transplantation NSG mouse with anti 5hmC (top) and Myeloperoxidase (MPO) (bottom). Note the absence of 5hmC in MPO^{+} leukemic cells which suggests that they are derived from transplanted Tet-deficient HSPCs. In all panels data presented as mean ± SEM. Statistically significant (*p<0.05, ** p<0.01, ***p<0.001). N.S. no significant change.

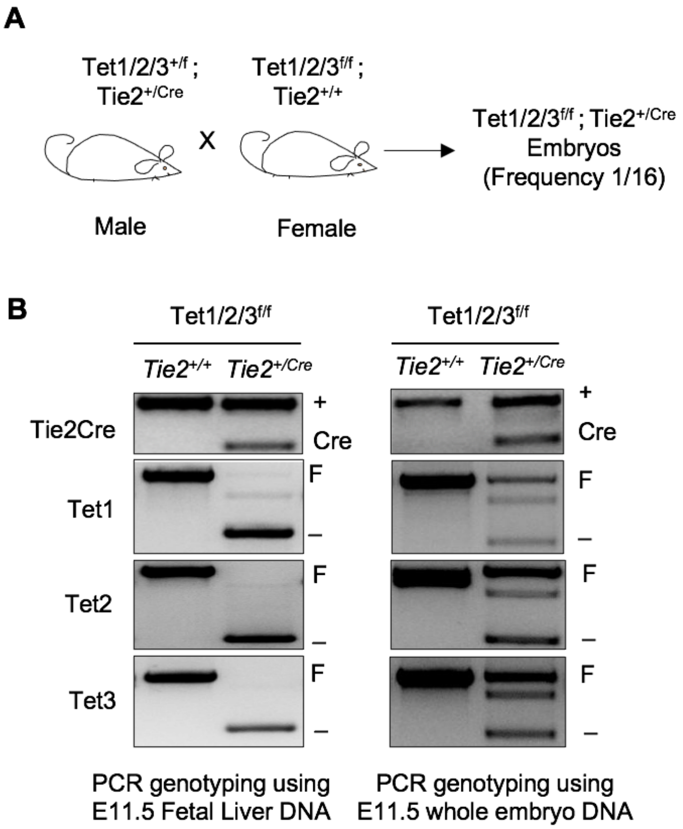

Fig. S4. Deletion of Tet enzymes in the endothelial lineage during embryogenesis using a *Tie2-Cre* strain. (A) Breeding strategy to generate *Tet1/2/3*$^{f/f}$*; Tie2*$^{+/Cre}$ embryos. **(B)** PCR genotyping of *Tie2-Cre*, *Tet1*, *Tet2* and *Tet3* alleles using DNA extracted from the E11.5 embryo proper or fetal liver.

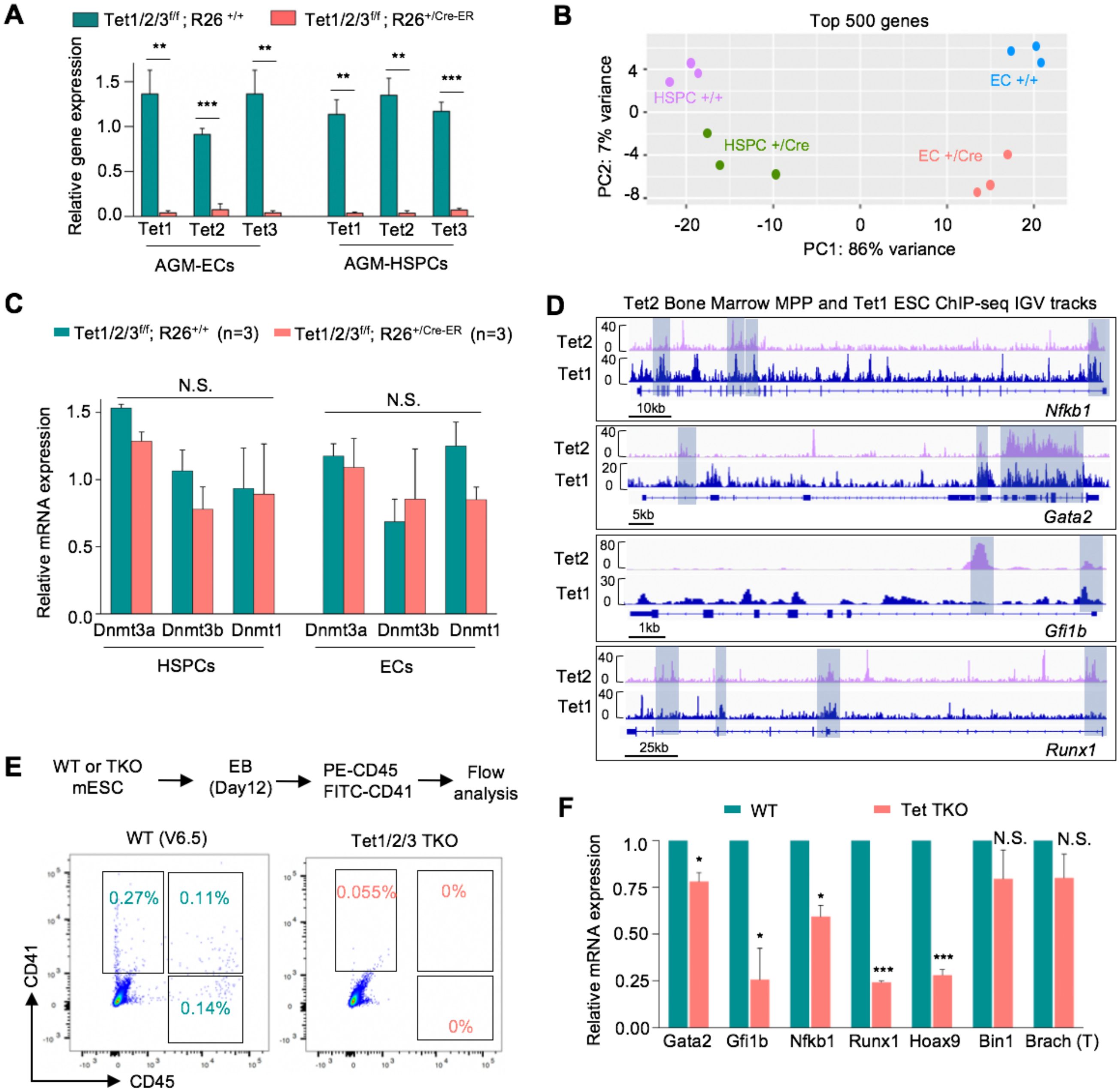

Fig. S5. Gene expression profile analysis of ECs and HSPCs in Tet-deficient AGMs. (A) Quantification of Tet1/2/3 mRNA levels in HSPCs and ECs (isolated from AGMs of E11.5 embryos that are tamoxifen treated at E7.5) by RT-qPCR confirming loss of expression of all three Tet enzymes. Data normalized to *Gapdh* expression. **(B)** Principal component analysis (PCA) of top 500 genes in each RNA-seq replicate of indicated genotypes. **(C)** Quantification of Dnmt1, Dnmt3a and Dnmt3b mRNA levels in HSPCs and ECs (isolated from AGMs of E11.5 embryos that are tamoxifen treated at E7.5) by RT-qPCR. Data normalized to *Gapdh* expression. **(D)** Genome browser tracks showing enrichment of Tet2 and Tet1 at genomic regions of selected deregulated genes using publicly available Tet2 bone morrow MPP (multipotent progenitors) and Tet1 ESC ChIP-seq datasets. **(E)** Quantification of $CD45^+$ and $CD41^+$ hematopoietic cells in day 12 wild type (WT) and Tet1/2/3 triple knockout (TKO) embryoid bodies (EBs) by flow cytometry. **(F)** Quantification of mRNA levels of indicated genes in day 12 wild type (WT) and Tet1/2/3 triple knockout (TKO) embryoid bodies (EBs) by RT-qPCR. Data normalized to *Gapdh* expression. For all panels data presented as mean ± SEM. Statistically significant (*p<0.05, ** p<0.01, ***p<0.001). N.S. not significant

A

Sample Name	Total no. of Cs analyzed, in millions	Methylated C's in CpG context	Unmethylated C's in CpG context	% mCpG	% mCHG	% mCHH
EC (+/+)	2315.9	80500621	40084327	66.8	0.5	0.7
EC (+/Cre)	1742.5	63240409	21550013	74.6	0.5	0.7
HSPC (+/+)	1982.8	70134948	33844676	67.5	0.4	0.7
HSPC (+/Cre)	1855.2	65699646	28100768	70.0	0.5	0.7

B

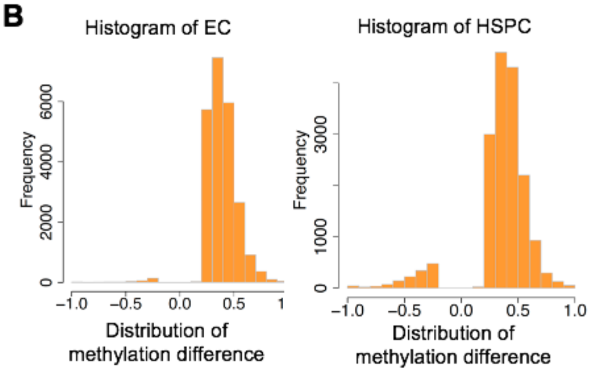

C

DMR to genes association (gene numbers shown)

Genomic regions	EC Cre vs. WT Hypermethylated genes	EC Cre vs. WT Hypomethylated genes	HSPC Cre vs. WT Hypermethylated genes	HSPC Cre vs. WT Hypomethylated genes
Promoter	1311	81	1114	97
5'UTR	248	6	212	13
3'UTR	499	3	446	34
Exon	1251	17	953	158
Intron	3847	78	3318	456
Distal Intergenic	4198	118	3138	518
**total**	**8041**	**297**	**6934**	**1234**

D

AGM EC DEGs with hypermethylated promoter DMRs

GO Term	-log of p-value
leukocyte differentiation	7.6
hemopoiesis	6.5
hematopoietic or lymphoid organ development	6.2
myeloid leukocyte activation	6.0
regulation of hemopoiesis	5.3
myeloid leukocyte differentiation	5.1

AGM EC DEGs with hypermethylated enhancer DMRs

GO Term	-log of p-value
cell activation	4.5
immune system development	3.7
myeloid leukocyte differentiation	3.6
hemopoiesis	3.6
leukocyte activation	3.4
hematopoietic or lymphoid organ development	3.4

E

Downregulated and hypermethylated genes in ECs (AGM)

9330133O14Rik	Ifi47	Rhof
Adgrg1	Il12a	Rhoh
Adgrg3	Itga2b	Rinl
Adh1	Kalrn	Sardh
Ank3	Kcnj5	Sh3tc2
Aprt	Kcnk6	Slc24a5
Bin2	Lat	Slc9a3r2
Caskin2	Lmna	Smim1
Cebpe	Lrmp	Smim3
D830046C22Rik	Ltf	Sncg
Dedd2	Ly6g6f	Sptb
Dok3	Lyz2	Srgn
Fam167b	Mdm1	Tbxa2r
Fam174b	Mfng	Tbxas1
Fermt3	Milr1	Tcf15
Gas2l1	Nlrx1	Tet3
Gimap1	Nrgn	Tmem40
Gimap5	P2rx1	Tnfrsf9
Gm5086	Pf4	Treml1
Gp9	Prkar2b	Trf
Grtp1	Prkcq	Tspan15
Gsap	Pttg1ip	Ubash3a
Gsg1	Rasal3	Unc119
H2-DMa	Rasgrp2	Unc13d
		Zdhhc14

GO Term	-log of p-value
cell activation	9.3
regulated exocytosis	8.1
blood coagulation	6.7
leukocyte degranulation	5.8
myeloid cell activation involved in immune response	5.7
myeloid leukocyte activation	5.6
myeloid leukocyte mediated immunity	5.6
hemopoiesis	3.9
regulation of hemopoiesis	3.8
hematopoietic or lymphoid organ development	3.7

Downregulated and hypermethylated genes in HSPCs (AGM)

1810055G02Rik	Fam117a	Ppcdc
AB124611	Fcgrt	Psap
AI662270	Fyb	Ptprcap
Akna	Galnt2	Rasal3
Aldh3b1	Glipr1	Rhoh
Apobec3	Gm128	Rin2
Apobr	Hcar2	Serinc3
Arhgap30	Hk3	Sh3tc2
Arhgef10l	Hmga2-ps1	Slc41a3
Armc7	Iffo1	Spn
Arrdc2	Igsf6	Sptb
Atf3	Lat2	Srgn
Atp8b4	Lcp1	Stap1
Bin1	Lgmn	Taok3
Bin2	Lrrc75a	Tbc1d22a
C1qa	Ly86	Tbc1d8
Ccdc88b	Map3k14	Tcirg1
Ccl3	Mcemp1	Tet3
Ccrl2	Mdm1	Tifab
Cd101	Mgl2	Tmem268
Cd300a	Milr1	Tnfaip2
Cd300c2	Mink1	Tnfaip8l2
Cd68	Mvp	Tnip1
Cebpe	Nfkb1	Trf
Coro2a	Ninj1	Trib1
Ctsb	Nostrin	Trio
Ctss	Olr1	Ubc
Ctsz	Optn	Unc13d
Dok3	Parvg	Wdr91
Fam105a	Phactr1	

GO Term	-log of p-value
myeloid leukocyte activation	16.4
myeloid leukocyte mediated immunity	15.0
leukocyte activation	14.5
leukocyte degranulation	14.2
myeloid cell activation involved in immune response	14.0
leukocyte activation involved in immune response	13.6
cell activation involved in immune response	13.5
cell activation	13.1
granulocyte activation	12.7
leukocyte mediated immunity	12.5

Fig. S6. DNA methylation profile analysis of ECs and HSPCs in Tet-deficient AGMs. (A) Summary of WGBS data. **(B)** Distribution of methylation difference at DMRs. Note majority of DMRs are 20-50% hypermethylated. **(C)** Association of genes to DMRs across different genomic regions. **(D)** Gene ontology (GO) analysis of DEGs with hypermethylated promoter or enhancer DMRs in ECs. **(E)** Down-regulated and hypermethylated DEGs in ECs or HSPCs (top) and their gene ontology analysis (bottom).

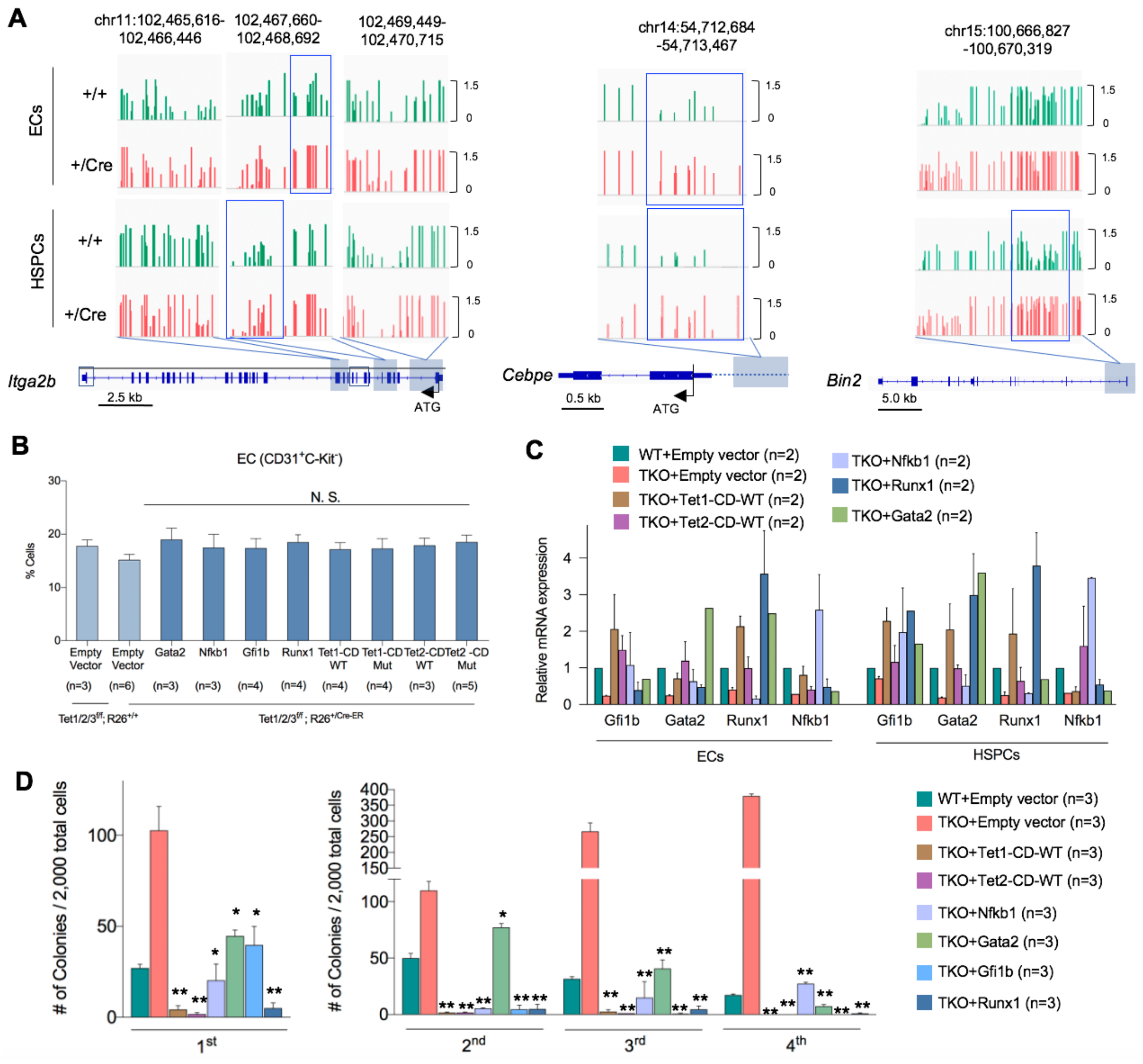

Fig. S7. DNA hypermethylation of hematopoietic regulators and rescue of hematopoiesis defects in Tet-deficient AGMs. (A) Representative genome browser tracks showing 5mC levels at selected down regulated master hematopoiesis transcription factors. Each vertical line on tacks represents a 5mC site. Blue frames indicate regions with more robust differences in methylation. **(B)** % ECs ($CD31^{+}c\text{-}Kit^{-}$) cells in AGMs of indicated genotypes quantified by flow cytometry and plotted. **(C)** Quantification of mRNA levels of indicated genes by RT-qPCR in ECs and HSPCs sorted from $Tet1/2/3^{f/f}$; $R26^{+/+}$(WT) or $Tet1/2/3^{f/f}$; $R26^{+/Cre\text{-}ER}$ (TKO) AGMs that were transduced with Tet catalytic domain (CD) or Nfkb1 or Gata2 or Runx1 or an empty vector. Data normalized to *Gapdh* expression. **(D)** Colony formation and replating capacity of HSPCs sorted from $Tet1/2/3^{f/f}$; $R26^{+/+}$(WT) or $Tet1/2/3^{f/f}$; $R26^{+/Cre\text{-}ER}$ (TKO) AGMs that were transduced with Tet catalytic domain (CD) or Nfkb1 or Gata2 or Runx1 or Gfi1b or an empty vector. In all panels data presented as mean ± SEM. Statistically significant (*p<0.05, ** p<0.01, ***p<0.001). N.S. not significant

Table S1. List of primers used in study

Name	Sequence (5'-3')
Tet1 Flox Genotyping For	AACTGATTCCCTTCGTGCAG
Tet1 Flox Genotyping Rev	TTAAAGCATGGGTGGGAGTC
Tet2 Flox Genotyping For	CATAGAGCTGTGTGTCCTGTTGTC
Tet2 Flox Genotyping Rev	GTGGAACATTACCTAAGCGAGAACC
Tet3 Flox Genotyping For	CTTAAGCAAAGCTCCCATGC
Tet3 Flox Genotyping Rev	ACCCCCAAATACCCAGAGTC
Tie2-Cre Genotyping common	AAA AATCAGCATTTTCAACAAA
Tie2-Cre Genotyping WT Rev	TTGGATTTTAGTCCCCTATCTGA
Tie2-Cre Genotyping Mut Rev	GTTTATTTACCGCCGTGTGTG
Rosa26-creER Genotyping 14930	TCTTGCGAACCTCATCACTC
Rosa26-creER Genotyping 01MR9020	AAGGGAGCTGCAGTGGAGTA
Rosa26-creER Genotyping 01MR9021	CCGAAAATCTGTGGGAAGTC
Bin1 RT-qPCR For	CAAGGCAAACTACAGGCTCATC
Bin1 RT-qPCR Rev	CCACGTTCATCTCCTCGAAC
Brach (T) RT-qPCR For	CTGGGAGCTCAGTTCTTTCGA
Brach (T) RT-qPCR Rev	GAGGACGTGGCAGCTGAGA
Gata2 RT-qPCR For	AAGCTGCACAATGTTAACAGG
Gata2 RT-qPCR Rev	CCTTTCTTGCTCTTCTTGGAC
Gfi1b RT-qPCR For	AAGAGCATAGCCAGAGTG
Gfi1b RT-qPCR Rev	TGATTGTGTTCCAGTCCAA
Hoax9 RT-qPCR For	ATGGCATTAAACCTGAACCG
Hoax9 RT-qPCR Rev	GTCTCCGCCGCTCTCATTC
Nfkb1 RT-qPCR For	GAAATTCCTGATCCAGACAAAAAC
Nfkb1 RT-qPCR Rev	ATCACTTCAATGGCCTCTGTGTAG
Tet1 RT-qPCR For	GCTGGATTGAAGGAAGAGGA
Tet1 RT-qPCR Rev	GTCTCCATGAGCTCCCTGAC
Tet2 RT-qPCR For	CCAAGACCAAGAAAGCAGCTCG
Tet2 RT-qPCR Rev	CCGAAAGCTGCGGTTGTGC
Tet3 RT-qPCR For	CCGTGACTGTGCTCTCAACT
Tet3 RT-qPCR Rev	CCATGAGTTCCCGGATAGAA
Runx1 RT-qPCR For	TCACTGGCGCTGCAACAA
Runx1 RT-qPCR Rev	TCTGCCGAGTAGTTTTCATCGTT
S100A8 RT-qPCR For	TGCGATGGTGATAAAAGTGG
S100A8 RT-qPCR Rev	GGCCAGAAGCTCTGCTACTC
S100A9 RT-qPCR For	CACAGTTGGCAACCTTTATG
S100A9 RT-qPCR Rev	CAGCTGATTGTCCTGGTTTG
S100A16 RT-qPCR For	GTATCCAAGCACAGCCTGGT
S100A16 RT-qPCR Rev	ATGAGCTTGTCAGCTGCCTT
Gata1 RT-qPCR For	TGCCTGTGGCTTGTATCA
Gata1 RT-qPCR Rev	TGTTGTAG GGTCGTTTGAC
Gata3 RT-qPCR For	CGGGTTCGGATGTAAGTCGA
Gata3 RT-qPCR Rev	GTAGAGGTTGCCCCGCAGT
Dnmt3a RT-qPCR For	GACTCGCGTGCAATAACCTTAG
Dnmt3a RT-qPCR Rev	GGTCACTTTCCCTCACTCTGG
Dnmt3b RT-qPCR For	CTCGCAAGGTGTGGGCTTTTGTAAC
Dnmt3b RT-qPCR Rev	CTGGGCATCTGTCATCTTTGCACC
Dnmt1 RT-qPCR For	GCTACCAGTGCACCTTTGGT
Dnmt1 RT-qPCR Rev	CAGAGGCAGCTTTTCTCCTG
Gapdh RT-qPCR For	GTGTTCCTACCCCCAATGTGT
Gapdh RT-qPCR Rev	ATTGTCATACCAGGAAATGAGCTT

Table S2. List of reagents used in study

REAGENT	SOURCE	IDENTIFIER
**Antibodies**		
Biotin rat anti-mouse CD31 clone: MEC13.3	BD Pharmingen™	Cat#553371; RRID: AB_394817
Streptavidin, Alexa Flour 488 conjugate	Invitrogen	Cat#S32354; RRID: AB_2315383
Alexa Flour 647 goat anti-rat IgG (H+L)	Invitrogen	Cat#A21247; RRID: AB_141778
Purified rat anti-mouse CD117 clone: 2b8	BD Biosciences	Cat#553352; RRID: AB_394803
CD117 (c-Kit) Monoclonal Antibody (2B8) APC	Thermo Fisher Scientific	Cat#17-1171-81; RRID: AB_469429
FITC anti-mouse CD31	Biolegend	Cat#102405; RRID: AB_312900
CD31 (PECAM-1) Monoclonal Antibody (390), PE	Thermo Fisher Scientific	Cat#12031181; RRID: AB_465631
PE/Cyanine7 anti-mouse CD31	Biolegend	Cat#102418; RRID: AB_830757
FITC Annexin V Apoptosis Detection Kit I	BD Biosciences	Cat#556547; RRID: AB_2869082
APC anti-mouse TER-119/Erythroid Cells	Biolegend	Cat#116212; RRID: AB_313713
PE anti-mouse CD45	Biolegend	Cat#103106; RRID: AB_312971
CD45.1 Monoclonal Antibody (A20), PE-Cyanine7	Thermo Fisher Scientific	Cat#25-0453-82; RRID: AB_469629
FITC anti-mouse CD45.2	Biolegend	Cat#109806; RRID: AB_313443
CD11b Monoclonal Antibody (M1/70), PE	Thermo Fisher Scientific	Cat#12-0112-85; RRID: AB_465549
CD45R (B220) Monoclonal Antibody (RA3-6B2), APC-eFluor 780	Thermo Fisher Scientific	Cat#470452-82; RRID: AB_1518810
CD3e Monoclonal Antibody (145-2C11), PerCP-Cyanine5.5	Thermo Fisher Scientific	Cat#450031-82; RRID: AB_1107000
Biotin Mouse Lineage Panel	BD Biosciences	Cat#559971; RRID: AB_10053179
CD41/CD61 Monoclonal Antibody (11C3), FITC	Invitrogen	Cat#MA5-28367; RRID: AB_2745330
Anti-5hmC	Active Motif	Cat# 39769; RRID:AB_10013602
Anti-TET1	EpiGentek	Cat# A-1020-100;
Anti-TET2	Abcam	Cat# ab124297; RRID: AB_2722695
Myeloperoxidase (MPO) antibody	Thermo Scientific	Cat# RB-373-A1
**Chemicals, Peptides, and Recombinant Proteins**		
Recombinant Mouse IL-3 Protein, CF	R&D Systems	Cat#403-ML-025
Mouse Flt-3 ligand	R&D Systems	Cat#427-FL
Recombinant Mouse SCF Protein, CF	R&D Systems	Cat#455-MC-020
Streptavidin/Biotin Blocking Kit	Vector Laboratories	Cat#SP-2002
Tamoxifen	Sigma	Cat#T5648-1G
Corn oil	Sigma	Cat#C8267
**Kits**		
MethoCult^{TM} GF	STEM CELL	Cat#M3434
XtremeGene 9 DNA transfection reagent	Roche	Cat#06365787001
Lenti-X-Concentrator	Takara	Cat#631231
LentiBlast Premium Transduction Enhancer	OZBiosciences	Cat#LBPX500
KAPA Mouse Genotyping Kit	KAPA Biosystems	Cat#KK7352
Click-iT™ EdU Alexa Fluor™ 647 Flow Cytometry Assay Kit	Invitrogen	Cat#C10424
AbC™ Total Antibody Compensation Bead Kit	ThermoFisher	Cat#A10497
**Experimental Models: Organisms/Strains/Cell Lines**		
NOD.Cg-Prkdcscid Il2rgtm1Wjl/SzJ (NSG) mice	Jackson lab	Cat#005557
B6.Cg-Tg(Tek-cre)12Flv/J (Tie2Cre) mice	Jackson lab	Cat#004128
B6;129-*Gt(ROSA)26Sor*$^{tm1(cre/ERT)Nat}$/J (Rosa26CreER) mice	Jackson lab	Cat#004847
Tet1/2/3 $^{F/F}$ mice	This paper	N.A.
Tet1/2/3 Triple Knockout (TKO) ESC	Reference 17	N.A.
**Recombinant DNA**		
FUW-HA-Nfkb1-2A-Tomato	This paper	N.A.
FUW-HA-Gfi1b-2A-Tomato	This paper	N.A.
FUW-HA-Tet1 catalytic domain (CD) wild type-2A-Tomato	This paper	N.A.
FUW-HA-Tet2 catalytic domain (CD) wild type-2A-Tomato	This paper	N.A.
FUW-HA-Tet1 catalytic domain (CD) mutant- 2A-Tomato	This paper	N.A.
FUW-HA-Tet2 catalytic domain (CD) mutant- 2A-Tomato	This paper	N.A.
FUW-mGata2-ires-GFP	This paper	N.A.
MSCV-Runx1-HA-IRES-GFP	This paper	N.A.
